# The Cultivable Bacterial Microbiota Associated to the Medicinal Plant *Origanum vulgare* L.: From Antibiotic Resistance to Growth-Inhibitory Properties

**DOI:** 10.3389/fmicb.2020.00862

**Published:** 2020-05-08

**Authors:** Lara Mitia Castronovo, Carmela Calonico, Roberta Ascrizzi, Sara Del Duca, Vania Delfino, Sofia Chioccioli, Alberto Vassallo, Iolanda Strozza, Marinella De Leo, Sauro Biffi, Giovanni Bacci, Patrizia Bogani, Valentina Maggini, Alessio Mengoni, Luisa Pistelli, Antonella Lo Nostro, Fabio Firenzuoli, Renato Fani

**Affiliations:** ^1^Department of Biology, University of Florence, Sesto Fiorentino, Italy; ^2^Department of Health Sciences, University of Florence, Florence, Italy; ^3^Department of Pharmacy, University of Pisa, Pisa, Italy; ^4^Giardino delle Erbe, Casola Valsenio, Italy; ^5^Research and Innovation Center in Phytotherapy and Integrated Medicine – CERFIT Careggi University Hospital, Florence, Italy

**Keywords:** antimicrobial compounds, medicinal plants, *Origanum vulgare*, endophytic bacteria, antibiotic resistance

## Abstract

The insurgence of antibiotic resistance and emergence of multidrug-resistant (MDR) pathogens prioritize research to discover new antimicrobials. In this context, medicinal plants produce bioactive compounds of pharmacological interest: some extracts have antimicrobial properties that can contrast different pathogens. For such a purpose, *Origanum vulgare* L. (Lamiaceae family) is a medicinal aromatic plant, whose essential oil (EO) is recognized for its antiseptic, antimicrobial and antiviral activities. The cultivable bacteria from different compartments (i.e., flower, leaf, stem and soil) were isolated in order to: (i) characterize the bacterial microbiota associated to the plant, determining the forces responsible for the structuring of its composition (by evaluation of cross inhibition); (ii) investigate if bacterial endophytes demonstrate antimicrobial activities against human pathogens. A pool of plants belonging to *O. vulgare* species was collected and the specimen chemotype was defined by hydrodistillation of its essential oil. The isolation of plant associated bacteria was performed from the four compartments. Microbiota was further characterized through a culture-independent approach and next-generation sequencing analysis, as well. Isolates were molecularly typed by Random Amplified Polymorphic DNA (RAPD) profiling and taxonomically assigned by 16S rRNA gene sequencing. Antibiotic resistance profiles of isolates and pairwise cross-inhibition of isolates on agar plates (i.e., antagonistic interactions) were also assessed. High level of diversity of bacterial isolates was detected at both genus and strain level in all different compartments. Most strains were tolerant against common antibiotics; moreover, they produced antagonistic patterns of interactions mainly with strains from different compartments with respect to that of original isolation. Strains that exhibited high inhibitory properties were further tested against human pathogens, revealing a strong capacity to inhibit the growth of strains resistant to several antibiotics. In conclusion, this study regarded the characterization of *O. vulgare* L. chemotype and of the bacterial communities associated to this medicinal plant, also allowing the evaluation of antibiotic resistance and antagonistic interactions. This study provided the bases for further analyses on the possible involvement of endophytic bacteria in the production of antimicrobial molecules that could have an important role in clinical and therapeutic applications.

## Introduction

Increasing antibiotic resistance, due to an overuse of antibiotics, and emergence of multidrug-resistant (MDR) pathogens prioritize research to discover new antimicrobials. The use of plants or their derivatives for prevention and treatment of various health ailments and infections is in practice from immemorial time (Sahoo et al., 2010): in fact, some plant extracts have antimicrobial properties able to affect different pathogens (Shityakova et al., 2019).

Microorganisms are naturally associated to plants in different ways (Reinhold-Hurek and Hurek, 2011). According to a widely used definition, “endophytic bacteria and fungi internally colonize the host tissues, sometimes in high numbers, without damaging the host or eliciting symptoms of plant disease” (Quispel, 1992). Most of the microorganisms inhabiting plants are important for the host’s development and health, despite, sometimes they could also be neutral (Mendes et al., 2013; Philippot et al., 2013).

Increasing knowledge indicates that microbes and/or their interactions with the host are responsible for the synthesis of natural products (Gunatilaka, 2006): for several medicinal plants, according to Köberl et al. (2013), “it is presumed that the plant-associated microbiome, especially the complex community of the endomicrobiome, is directly or indirectly involved in the production of bioactive phytochemicals”. Indeed, endophytic microorganisms play an important role in searching for natural bioactive compounds, with potential use in the health sector and in drug discovery (Lam, 2007). It is well known that, within plant diversity, a distinctive microbiota is harbored by medicinal plant species due to their structurally divergent and unique secondary metabolites (Qi et al., 2012).

*Origanum vulgare* L. is a medicinal aromatic plant belonging to the *Lamiaceae* family, which comprises many essential oil (EO) producing species. *O. vulgare* is morphologically considered to be one of the most variable species within the genus *Origanum*. Moreover, its variability is also exhibited in the chemotype, which is highly influenced by the plant growth environment. The most represented compounds in oregano EO are phenolic monoterpenes, such as carvacrol and thymol (Werker et al., 1985; De Mastro et al., 2017), followed by linalool and linalyl acetate (De Mastro et al., 2017). Thymol and carvacrol have been reported to exert a substantial inhibitory activity against microbes (Lambert et al., 2001): in fact, it is known that the antibacterial properties of EO are mainly due to its phenolic compounds (Cosentino et al., 1999). Other published studies report EO compositions rich in sesquiterpene hydrocarbons, such as germacrene D and β-caryophyllene (Mockute et al., 2001). These two compounds are major components also in other medicinal plants EOs, such as those of *Cedrus libani* A. Rich. and *Artemisia vulgaris* L., and they are known to act as antimicrobial, anesthetic and anti-inflammatory compounds, with cytotoxic activity against several cancer cell lines (Saab et al., 2018; Malik et al., 2019). According to Checcucci et al. (2017), the plant species (medicinal or aromatic ones) that produce EOs could represent a suitable model for “testing the hypothesis of an effect of the endophyte-microbiome dichotomy on the production of EOs and, consequently, of a colonization of plant tissues by bacteria resistant to these oils,” as well as the role of plant-bacteria interaction in the modulation of medicinal plant secondary metabolism (Maggini et al., 2019a, b, 2020).

In this work, the chemotype of *O. vulgare* EO was determined and the cultivable bacteria from different compartments (flower, leaf, stem and soil) were isolated. The main goals were (i) to characterize the bacterial microbiota associated to the plant, determining the forces responsible for the structuring of its composition (by evaluation of cross inhibition); (ii) to investigate if bacterial endophytes demonstrate antimicrobial activities against human pathogens.

## Materials and Methods

### Hydrodistillation of the Essential Oil

A pool of three plants of *O. vulgare* L. cultivated in an open-air common garden at the “Giardino delle Erbe - Augusto Rinaldi Ceroni,” Casola Valsenio (Ravenna, Italy), were collected in July 2018. 1 kg of above ground tissues was distilled. The steam distillation of EO was carried out at the Giardino delle Erbe (Casola Valsenio, RA, Italy), following the method described in Hanci et al. (2003).

### Determination of the Essential Oil Composition

The pure EO was diluted at 5% in HPLC-grade *n*-hexane and then injected in the GC/EI-MS apparatus. The GC/EI-MS analyses were performed with a Varian CP-3800 apparatus equipped with a DB-5 capillary column (30 m × 0.25 mm i. d., film thickness 0.25 μm) and a Varian Saturn 2000 ion-trap mass detector. The oven temperature was programmed rising from 60 to 240°C at 3°C/min. The set temperatures were as follows: injector temperature, 220°C; transfer-line temperature, 240°C. The carrier gas was He, at 1 ml/min flow. The injection volume was set at 1 μl. The acquisition was performed with the following parameters: full scan, with a scan range of 35–300 *m/z*; scan time: 1.0 s; threshold: 1 count. The identification of the constituents was based on the comparison of their retention times (t_*R*_) with those of pure reference samples and their linear retention indices (LRIs), which were determined relatively to the t_*R*_ of a series of *n*-alkanes. The detected mass spectra were compared with those listed in the commercial libraries NIST 14 and ADAMS, as well as in a homemade mass-spectral library, built up from pure substances and components of commercial essential oils of known compositions, and in MS literature data (Masada, 1976; Jennings and Shibamoto, 1982; Davies, 1990; Swigar and Silverstein, 1992; Adams, 1995; Adams et al., 1997).

### Sampling of *O. vulgare* and Plant-Associated Bacteria Extraction

From the same pool of plants of *O. vulgare* used for hydrodistillation, plant anatomical parts (i.e., flower, leaf, stem) and soil near the plant roots (bulk soil) were separately collected and treated as independent samples. Flowers were grouped and pooled, as well as the stems and leaves. 1 g of fresh tissue from each pool was surface sterilized with 1% v/v HClO in a sterile 50 ml tube at room temperature in order to remove the epiphytic bacteria, as described in Chiellini et al. (2014). After this, samples were washed three times with sterile water, and then they were pottered in a sterile mortar with the addition of 2 ml of 0.9% w/v NaCl sterile solution. Water used for the last wash was plated on TSA to verify sterility of sample external surface. Bulk soil was treated separately at room temperature for 1 h with 20 ml of 10 mM Mg_2_SO_4_ in a 50 ml sterile tube under shaking, to allow the detachment of bacteria from soil particles.

### Extraction of Genomic DNA and NGS Sequence Analysis

Genomic DNA was extracted from flower, leaf and stem samples using PowerLyzer^®^ PowerSoil^®^ DNA Isolation Kit (MO BIO laboratories, Inc., Carlsbad, CA, United States) following the manufacturer’s instruction. Hypervariable regions of bacterial 16S rRNA gene (V1–V9) (Chakravorty et al., 2007; Petrosino et al., 2009) were used as molecular markers for bacterial identification in HTS analysis (Huse et al., 2008). In this study, the V3–V4 regions were sequenced using the primer 341F and 805R (Herlemann et al., 2011) according to the protocol reported in the 16S Metagenomic Sequencing Library Preparation protocol from Illumina (Kozich et al., 2013). Library preparation and demultiplexing were performed following Illumina’s standard pipeline (Caporaso et al., 2012). Libraries were sequenced in a single run using Illumina MiSeq technology with pair-end sequencing strategy with MiSeq Reagent Kit v3. Library construction and sequencing were performed by an external company (BMR Genomics, Padua, Italy). Sequence files have been submitted in the NCBI sequence read archive (SRA) and are available under the accession PRJNA606513.

### Amplicon Sequence Variant Inference

Sequences were clustered into ASVs using the DADA2 pipeline reported at https://benjjneb.github.io/dada2/tutorial.html (Callahan et al., 2016). PCR primers were removed with cutadapt (Martin, 2011) with default settings. Sequences were discarded if the software did not detect one or both primers (–discard-untrimmed option). To ensure that sequences were paired after the trimming process the ‘-pair-filter = any’ option was used. Sequences were then filtered using the ‘filterAndTrim’ function of DADA2 with a maximum error rate of 2 and a fixed length of 270 and 200bp for forward and reverse reads, respectively. Trimmed sequences were used for error rate estimation (the ‘learnErrors’ function with default parameters). Finally, sequences were denoised and merged, and variants were inferred using the DADA2 algorithm. Taxonomic annotation was performed after chimera removal using DECIPHER package (Murali et al., 2018) (version 2.0) with the Silva training set 132 (Quast et al., 2013). Tables produced by the DADA2 pipeline were imported into phyloseq (through the phyloseq R package version 1.22.3), ASVs assigned to chloroplasts and mitochondria have been excluded, and bacterial communities of the three compartments were analyzed.

### Count and Isolation of Cultivable Bacteria

Tissue extracts and soil suspensions were diluted (10^–1^, 10^–3^, 10^–5^, 10^–7^) and 100 μl of each dilution were plated on tryptic soy agar (TSA) medium (Bio-Rad) in triplicate. Plates were incubated at 30°C and the CFU (colony forming units) were counted after 48 h. For each compartment about 25 different colonies were randomly chosen; then, they were streaked on TSA medium and grown at 30°C for 48 h.

### Bacterial Strains and Growth Conditions

The bacterial collection is represented by a panel of 97 isolates from different compartments of *O. vulgare* L. plants (24 isolates from flower compartment, 24 from leaf, 25 from stem and 24 from bulk soil). Isolates are referred to as OV followed by F, L, S or T for flower, leaf, stem and bulk soil districts, respectively, and numerated. Bacterial isolates were grown on TSA medium for 48 h at 30°C.

### Random Amplified Polymorphic DNA Analysis

Cell lysates were prepared by processing a single isolated colony resuspended in 20 μl of sterile dH_2_O, by thermal lysis (95°C for 10 min), followed by cooling on ice for 5 min. RAPD profiles of the bacterial isolates were obtained using the primer 1253 (Table 1). The reaction mix was performed in a 25 μl-volume with 1× DreamTaq Buffer, 200 μM dNTPs, 500 ng of primer 1253, 1 U of DreamTaq DNA Polymerase (Thermo Scientific) and 2 μl of thermal lysate used as template. The PCR cycling adopted was set up in a Bio-Rad T100 thermal cycler as follows: 90°C for 1 min, 95°C for 95 s followed by 45 cycles of 95°C for 30 s, 36°C for 1 min, 75°C for 2 min, then 75°C for 10 min and finally 60°C for 10 min. Amplicons were visualized through a 2% w/v agarose gel electrophoresis. The assignment of different isolates to the same haplotype group was determined on the basis of the fingerprint pattern of each RAPD product, comparing them for the presence/absence of bands. For haplotypes represented by more than one isolate derived from the same compartment, a single bacterial isolate was randomly chosen as representative strain for that RAPD haplotype.

**TABLE 1:** Primers used in this work.

Primer	Sequence (5′ > 3′)	Amplicon	References
P0	GAGAGTTTGATCCTGGCTCAG	16S rDNA	Di Cello and Fani, 1996
P6	CTACGGCTACCTTGTTACGA		
1253	GTTTCCGCCC	RAPDs	Mori et al., 1999

### 16S rRNA Gene Sequences

For each recognized RAPD haplotype, the 16S rRNA coding gene sequence was used for the taxonomic affiliation of the bacterial isolates. Amplification of 16S rDNA gene was obtained in a total volume of 20 μl, containing 1× DreamTaq Buffer, 250 μM dNTPs, 0.6 μM of primers P0 and P6 (Table 1), 2 U of Dream Taq DNA Polymerase (Thermo Scientific), and 1 μl of thermal lysate used as template. Samples were incubated for 3 min in a thermal cycler (Bio*-*Rad T100) at 95°C, then amplification was achieved with 30 cycles of 30 s at 95°C, 30 s at 50°C, and 1 min at 72°C, with a final extension at 72°C for 10 min. Amplicons were analyzed through a 0.8% w/v agarose gel electrophoresis. Sequencing of the 16S rDNA amplicons was performed by the Microsynth Seqlab (Germany). Each sequence was submitted to Gene Bank and the accession numbers from No MN811044 to No MN811099 (Supplementary Table S1).

### Phylogenetic Tree Analysis

Taxonomic affiliation of the strains was determined through the analysis of 16S rRNA gene sequences obtained. They were aligned to those of type strains retrieved from the Ribosomal Database Project (RDP) (Cole et al., 2014) using BioEdit (Hall, 1999). The obtained alignment was then used to build a phylogenetic tree through MEGA X (Kumar et al., 2018) for each genus, applying the Neighbor-Joining algorithm with a 1000-bootstrap resampling.

### Antibiotics Resistance Profile of Cultivable Bacteria Associated to *O. vulgare*

Evaluation of antibiotic resistance was performed by streaking each strain on TSA medium containing different concentration of selected antibiotics, according to Mengoni et al. (2014). The chosen antibiotics were chloramphenicol, ciprofloxacin, rifampicin, streptomycin, kanamycin, and tetracycline (Table 2). After growing on TSA medium for 48 h at 30°C, a colony of each strain was suspended in 100 μl saline solution (0.9% w/v NaCl), streaked on TSA medium supplemented with different antibiotics and then incubated at 30°C for 48 h. The following antibiotic concentrations (μg/ml) were tested: chloramphenicol (1-2.5-5-10-25-50); ciprofloxacin, streptomycin and kanamycin (0.5-1-2.5-5-10-50); rifampicin (5-10-25-50-100); tetracycline (0.5-1.25-2.5-5-12.5-25). The different growth levels were indicated as follows: complete growth (+ +), strong (+), weak (+ −), very weak (+ −), and absence of growth (−), and MIC (Minimal Inhibiting Concentration) values were identified.

**TABLE 2:** Classes and targets of the antibiotics used in this work.

Antibiotic	Class	Target
		
Chloramphenicol	Phenicols	Ribosome
Ciprofloxacin	Fluoroquinolones	Topoisomerases
Kanamycin	Aminoglycosides	Ribosome
Rifampicin	Ansamycins	RNA polymerase
Streptomycin	Aminoglycosides	Ribosome
Tetracycline	Tetracyclines	Ribosome

### Analysis of Antagonistic Interactions Through Cross-Streaking

Bacterial strains isolated from each compartment of *O. vulgare* plants were tested for cross antagonism, using representatives of each RAPD haplotype. Antagonistic interactions were assayed between each compartment of *O. vulgare* within-niche and cross-niche. Tester strains were firstly streaked across one half of a TSA plate and grown at 30°C for 48 h in order to allow the possible production of antimicrobial compounds. Target strains were then streaked perpendicularly to the tester strain and plates were incubated at 30°C for further 48 h. Additionally, target strains were grown at 30°C for 48 h in the absence of the tester, as growth control. The antagonistic effect was evaluated as the absence or reduction of the target strains growth compared to their growth in the absence of the tester strain. The different inhibition levels were indicated with numbers from 0 to 3 as follows: complete (3, red), strong (2, orange), weak (1, salmon), and absence (0, white) of inhibition (Maida et al., 2016).

### Inhibition of Human Pathogens by *O. vulgare* Associated Bacteria

A panel of selected strains of *O. vulgare* for which high antagonistic interactions were evidenced, was tested against 46 pathogenic strains: 10 for *Staphylococcus aureus*, 10 for Coagulase-negative staphylococci, 10 for *Pseudomonas aeruginosa*, 6 for *Klebsiella pneumoniae* characterized by their resistance to multiple antibiotics (as reported in Table 3), and 10 of a *Burkholderia cepacia* complex (BCC) collection, MDR BCC bacteria able to resist to different antibiotic classes (e.g., polymyxins, most of beta-lactams and aminoglycosides) (Saiman et al., 2003). The bacterial strains were isolated from different sources (hospital devices, foods, patients, healthy subjects, environment), as shown in Table 3. As reported in the BCCM/LMG bacteria collection database, origins of the BCC strains classified as environmental are the following: LMG1222, onion; LMG17588, soil; LMG19182, pea rhizosphere; LMG19230, wheat roots. Each strain was maintained at −80°C under glycerol (25%, v/v) stock, cultured in Brain Heart Infusion Broth (Thermo Scientific) for 24 h at 37°C, then streaked on TSA, and incubated at 37°C for 24 h before the use. The isolated bacterial strains (*S. aureus*, CoNS, *P. aeruginosa, K. pneumoniae*) were provided from the Applied Microbiology laboratory (Health Sciences Dept., University of Florence, Italy), while the standard bacteria *Staphylococcus aureus* ATCC 25923, *Pseudomonas aeruginosa* ATCC 27853 and *Klebsiella pneumonia* ATCC 700603 were obtained from Thermo Fisher Diagnostics S.p.A. The antimicrobial activity was evaluated through the cross-streak method, as previously described, except for the incubation temperature of target strains: 48 h at 37°C for CoNS (Coagulase-negative staphylococci) and 24 h at 37°C for all the other pathogens. The degree of antagonistic effect was evaluated by measuring the inhibition or reduction zone and the results were presented with numbers from 0 to 3 as previously described. The sum of numbers of each bacterial strain represents the total score of inhibition (TSI) ability against human pathogens. TSI was scored as absence of activity (TSI = 0), very low activity (TSI = 1–10), low activity (TSI = 11–20), strong activity (TSI = 21–27), and very strong activity (TSI = 28–30) (Maida et al., 2016).

**TABLE 3:** Pathogenic strains used in the study and related antibiotic resistance profiling.

Pathogens	Strain code	Origin	Antibiotic resistance profiling
*S. aureus*	ATCC 25923	−	P, NA
	2668	F	AMP, P, DA, TE, E, K, NA
	2749	HS	VA, TEC, DAP
	3709	F	DA, TE, E, K, NA
	3710		DA, TE, E, K, NA
	4070		P, DA, TE, E, CIP, LEV, DAP
	4168		AMP, P, DA, SXT, TE, NA
	4302		P, FOX, SXT, DAP
	4691	P	P, FOX, E, CN, CIP, LEV, DAP
	4708		P, FOX, CN, VA, DAP
*S. haemolyticus*	5284	HD	P, TE, E, CN, FD
	5285		E, CN, CIP, LEV, FD
	5383		P, FOX, TE, E, CN
	5396	HS	P, FOX, SXT, CN, FD
*S. epidermidis*	5318	HS	P, E, CN, FD
	5321		P, E, CN, AK, FD
	5323		P, TE, TIG, E, CN
	5377	HD	P, TE, E, TEC
	5403	HS	P, E, TIG, TE
	5419	F	FOX, DA, CIP, LEV, SXT, TIG
*P. aeruginosa*	ATCC 27853	−	FOX, K
	4177	HD	AK, TOB, CIP, LEV, CAZ, FEP, MEM, IPM, ATM, PRL, TZP
	4189		AK, TOB, CIP, LEV, CAZ, FEP, MEM, IPM, PRL, TZP
	5234		AK, CAZ, ATM, TZP, PRL, FEP, CN, IPM, MEM, LEV, CIP, TOB
	5245		CAZ, ATM, PRL, FEP, CN, LEV, CIP, IPM, MEM, TOB
	5246		CAZ, ATM, TZP, PRL, FEP, CN, IPM, MEM, LEV, CIP, TOB
	5255		AK, ATM, CN, FEP, TOB
	5139		CIP, CN, FEP, LEV, TOB
	5009		ATM, CAZ, CIP, CN, FEP, IPM, LEV, MEM, PRL, TOB, TZP
	5236		AK, ATM, CAZ, CIP, FEP, IPM, LEV, MEM, TOB
*K. pneumoniae*	ATCC 700603	−	CAZ, AMP, ATM, PRL, TE
	4409	P	AK, AMX, FEP, CTX, CAZ, CIP, ETP, IPM, MEM, TZP, SXT, FFL, CL, TIG
	4412		AMX, FEP, CTX, CAZ, CIP, ETP, IPM, MEM, TZP, SXT, FFL, TIG
	4417		AK, AMX, FEP, CTX, CAZ, CIP, ETP, IPM, MEM, TZP, SXT, FFL, CL, TIG
	4420		AK, AMX, FEP, CTX, CAZ, CIP, IPM, MEM, TZP, SXT, FFL, CL, CN
	4422		AK, AMX, FEP, CTX, CAZ, CIP, ETP, IPM, MEM, TZP, SXT, FFL
*B. cepacia*	FCF3	CF	
*B. cenocepacia*	FCF23		
*B. multivorans*	LMG13010		
*B. ambifaria*	LMG_16656		
*B. cenocepacia*	LMG_21462		
*B. cenocepacia*	LMG_24506		
*B. cepacia*	LMG_1222	E	
*B. multivorans*	LMG_17588		
*B. ambifaria*	LMG_19182		
*B. cenocepacia*	LMG_19230		

*F = food, HD = hospital devices, HS = healthy subjects, P = patients, CF = Cystic Fibrosis, E = environment. Amikacin (AK), Amoxicillin (AMX), Ampicillin (AMP), Aztreonam (ATM), Cefepime (FEP), Cefotaxime (CTX), Cefoxitin (FOX), Ceftazidime (CAZ), Ciprofloxacin (CIP), Clindamycin (DA), Colistin (CL), Daptomycin (DAP), Ertapenem (ETP), Erythromycin (E), Florfenicol (FFL), Fusidic Acid (FD), Gentamicin (CN), Imipenem (IPM), Kanamycin (K), Levofloxacin (LEV), Meropenem (MEM), Nalidixic Acid (NA), Penicillin G (P), Piperacillin (PRL), Piperacillin/Tazobactam (TZP), Sulfamethoxazole/Trimethoprim (SXT), Teicoplanin (TEC), Tetracycline (TE), Tigecycline (TIG), Tobramycin (TOB), Vancomycin (VA).*

## Results

### Chemical Characterization of the *O. vulgare* L. Essential Oil and Chemotype Attribution

The complete composition of the EO hydrodistilled from the analyzed *O. vulgare* specimen is reported in Supplementary Table S2. Terpene hydrocarbons dominated the composition, since they were detected in a relative amount accounting for over 90% of the total. Sesquiterpene hydrocarbons, in particular, added up to over 70% of the total: among them, germacrene D and β-caryophyllene were the most abundant, exhibiting relative abundances of 29.4 and 19.2%, respectively. The plant was, thus, a germacrene D/β-caryophyllene chemotype. The oxygenated terpenes were far less represented, accounting only for the remaining 8.6%: among them, only 4-terpineol, caryophyllene oxide and spathulenol exhibited relative concentrations higher than 1%.

### Characterization of the Plant Microbiota

**FIGURE 1:**
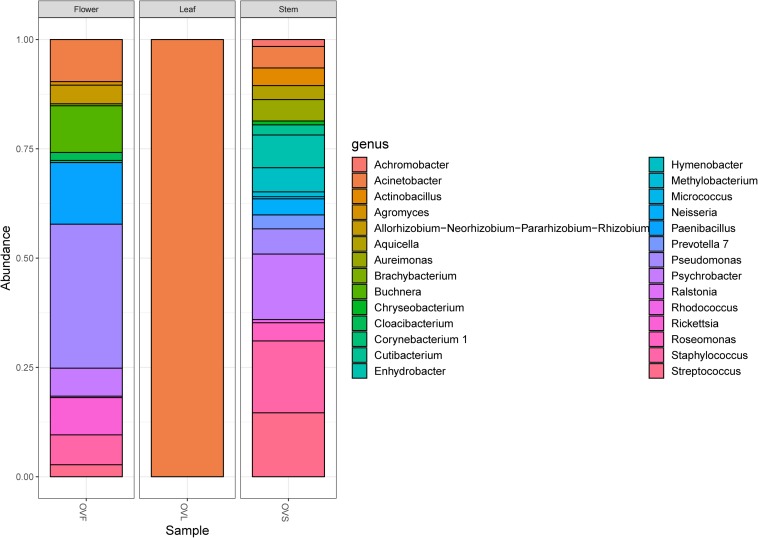
Bar plot showing the relative abundances of bacterial genera in each sample. OVF = flower, OVL = leaf, OVS = stem compartment.

Bacterial communities of plant samples of the same compartments from which the EO of *O. vulgare* plants was obtained (i.e., flower, leaf, and stem) were examined through NGS. Quality filtering steps obtained a total of 53 ASVs, and representative sequences for each ASV were classified into 28 genera belonging to 4 phyla according to the Silva database, with a different distribution between leaves (with only one genus, *Acinetobacter*, found) and the other two compartments. The majority of the ASVs (62.26%) belong to *Proteobacteria* phylum, followed by *Firmicutes* (15.09%), *Actinobacteria* (11.32%), and *Bacteroidetes* (11.32%). Data obtained are shown in Figure 1.

### Isolation of Cultivable Bacteria

Bacteria extracted from flower, leaf, stem of *O. vulgare* plants and bulk soil were plated as described in Materials and Methods. Data obtained revealed that the highest bacterial titers were detected in the soil (5.1 × 10^5^), while the lowest CFU/g value was represented by the leaves (7 × 10^2^); flower and stem compartments had 2.16 × 10^5^ and 8.5 × 10^3^ CFU/g, respectively.

### Strain-Level Profiling of Isolates

To investigate the structure of the culturable microbiota and to define the haplotypes (i.e., individual genotypes) present, a RAPD analysis was performed assuming that isolates sharing the same RAPD fingerprinting correspond to the same strain (Chiellini et al., 2014 and references therein): a total of 62 different RAPD haplotypes was identified, corresponding to 62 different bacterial strains. The distribution of the RAPD haplotypes within the different compartments is shown in Supplementary Table S3. The highest number of haplotypes was detected in the stem compartment (19 haplotypes out of 25 bacterial isolates), while the soil compartment exhibited the lowest number of haplotypes (12 haplotypes out of 24 isolates) as shown in Table 4. Notably, a very low number of haplotypes was shared between the different compartments: indeed, only 2 strains were shared by the leaf and stem compartments.

**TABLE 4:** Distribution of bacterial haplotypes, species, and genera detected in the four districts of *O. vulgare* plant.

		Flower (OVF)	Leaf (OVL)	Stem (OVS)	Soil (OVT)	Total	%
N. of isolates		24	24	25	24	97	/
N. of haplotypes		16	17	19	12	62	/
N. of species		11	13	13	9	32	/
N. of genera		8	8	8	6	19	/
N. of shared haplotypes	Flower	−	0	0	0	2	3.2
	Leaf	−	−	2	0		
	Stem	−	−	−	0		
	Soil	−	−	−	−		
N. of shared species	Flower	−	1	3	2	9	28
	Leaf	−	−	5	4		
	Stem	−	−	−	5		
	Soil	−	−	−	−		
N. of shared genera	Flower	−	2	3	1	7	37
	Leaf	−	−	5	0		
	Stem	−	−	−	1		
	Soil	−	−	−	−		

### Taxonomic Assignment of Strains

**FIGURE 2:**
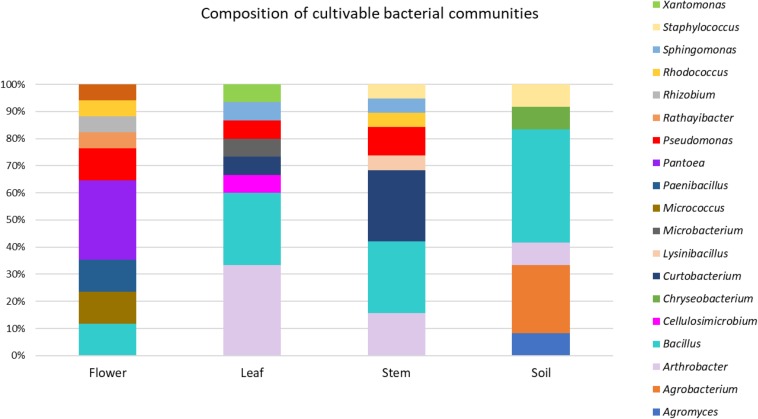
Composition of cultivable bacterial communities of *O. vulgare*. Number of genera for each district: flower (8), leaf (8), stem (8), soil (6).

The taxonomic identification of strains from RAPD haplotyping by 16S rRNA gene sequencing is summarized in Figure 2.

**FIGURE 3:**
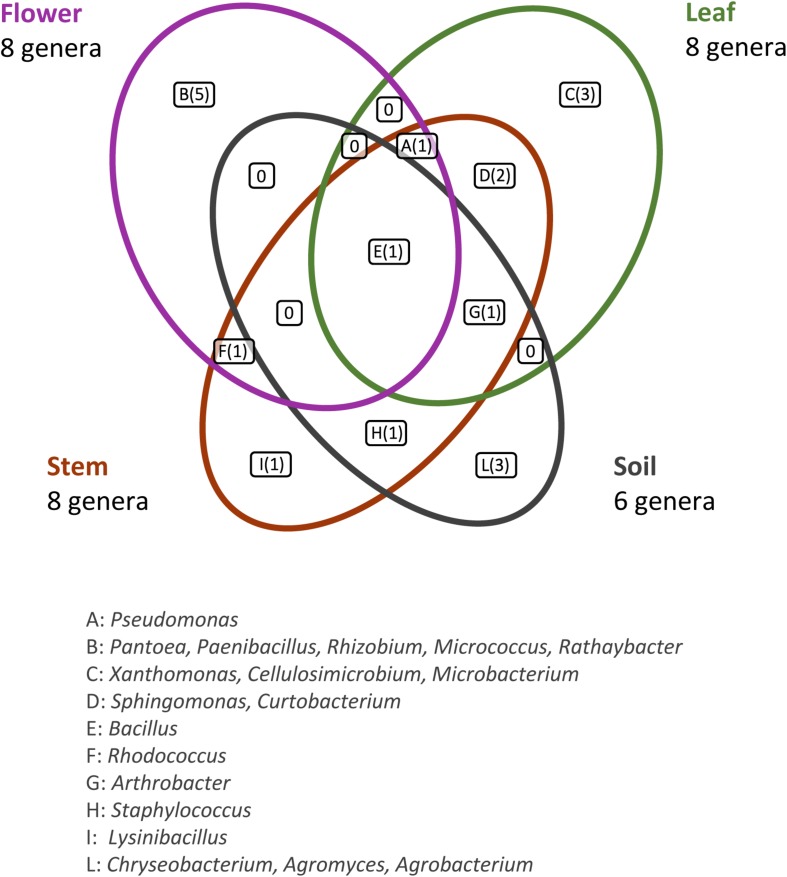
Bacterial genera shared by the different anatomical parts of the plant and in the bulk soil. The number of different genera identified is reported in brackets.

This analysis revealed that: (i) strains were affiliated to 19 different bacterial genera (42.1% Gram negative, 57.9% Gram positive); (ii) the majority of the 16S rDNA gene sequences was affiliated to the genus *Bacillus* (28.12%); (iii) *Arthrobacter* was the second most highly represented genus (14.06%); (iv) seven genera (*Pseudomonas, Sphyngomonas, Curtobacterium, Arthrobacter, Bacillus, Staphylococcus, Rhodococcus*) were shared among compartments, however just the genus *Bacillus* was shared by all the four districts. Moreover, a total of 12 genera were represented in only a single district, such as *Pantoea, Rhizobium, Paenibacillus, Micrococcus*, and *Rathayibacter*, which were detected only in the flower compartment (Figure 3).

For each genus a 16S rRNA gene phylogenetic tree was constructed as described in Materials and Methods. The analysis of the obtained phylogenetic trees (shown in Supplementary Material from Figures S1 to Figure S18) allowed to affiliate the 62 strains to 32 (possible) different species. The 28.1% of species resulted to be shared by the flower, leaf, stem, and soil compartments. Only one species (i.e., *Bacillus megaterium*) resulted to be shared between all the four districts. The compartments with the highest number of species were leaf and stem districts (for both of them 13 different species were identified).

### Antibiotics Resistance Profiles

The antibiotic resistance profiles of bacteria associated to the four *O. vulgare* compartments were investigated, as described in Materials and Methods. The Minimal Inhibitory Concentration (MIC) of each antibiotic was evaluated and the values are reported in Table 5. The 45.16% of the *O. vulgare* associated bacteria showed MIC value for the antibiotic streptomycin < 2.5 μg/ml, and the 37.09% showed a very high MIC level (50 μg/ml). Regarding the antibiotic kanamycin the 41.93% revealed MIC value at the maximum concentration used or more. In particular, a very high tolerance to streptomycin and kanamycin, even to the highest concentrations used (MIC values > 50 μg/ml), was revealed by some strains associated to the bulk soil and leaves. MIC value for tetracycline was evaluated at low concentrations (< 2.5 μg/ml) for the majority of the strains (83.87%). Some strains showed the capacity to grow in presence of low concentrations of chloramphenicol and ciprofloxacin, and only a few strains (12 out of 62) demonstrated to tolerate the lowest concentrations (5 μg/ml) of rifampicin as well. Moreover, the 89.40% of the bacteria associated to the stem were almost totally inhibited by streptomycin, even at the lowest concentration. The different antibiotic resistance profiles seemed to not correlate with the species affiliation and/or the districts from whom bacteria were isolated. Nonetheless, just in case of streptomycin resistance of *Arthrobacter* strains, those isolated from the stem were much more sensitive in comparison to those ones associated to the leaves. Overall, strains associated to the bulk soil resulted tolerant to the great majority of the antibiotics tested, even at the highest concentrations used.

**TABLE 5:** Antibiotic resistance assay of *O. vulgare* associated bacteria.

Compartment	Strain	Taxonomy	MIC (μg/ml)					
			
			Str.	Tet.	Cip.	Kan.	Chl.	Rif.
T	OVT1	*Agrobacterium*	>50	2.5	<0.5	50	25	10
T	OVT8	*Agrobacterium*	>50	2.5	<0.5	50	25	10
T	OVT17	Agrobacterium	>50	2.5	<0.5	50	25	10
T	OVT2	Agromyces	>50	>25	50	>50	10	<5
S	OVS8	*Arthrobacter*	<0.5	<0.5	5	50	<1	<5
S	OVS18	*Arthrobacter*	<0.5	<0.5	5	>50	< 1	<5
S	OVS23	*Arthrobacter*	<0.5	1.25	2.5	50	<1	<5
L	OVL1	*Arthrobacter*	>50	< 0.5	10	>50	< 1	<5
L	OVL3	*Arthrobacter*	50	<0.5	5	> 50	< 1	<5
L	OVL4	*Arthrobacter*	50	<0.5	2.5	>50	< 1	<5
L	OVL20	*Arthrobacter*	50	<0.5	5	> 50	< 1	<5
L	OVL22	*Arthrobacter*	50	<0.5	5	> 50	< 1	<5
T	OVT23	*Arthrobacter*	50	<0.5	5	> 50	2.5	<5
L	OVL7	*Bacillus*	<0.5	<0.5	<0.5	<0.5	2.5	<5
L	OVL8	*Bacillus*	5.0	<0.5	<0.5	1	5	<5
S	OVS6	*Bacillus*	<0.5	<0.5	<0.5	<0.5	10	<5
S	OVS10	*Bacillus*	<0.5	2.5	<0.5	<0.5	2.5	<5
S	OVS21	*Bacillus*	<0.5	2.5	<0.5	<0.5	2.5	<5
F	OVF22	*Bacillus*	2.5	2.5	<0.5	<0.5	<1	<5
L	OVL9	*Bacillus*	2.5	1.25	<0.5	1	<1	<5
S	OVS26	*Bacillus*	2.5	1.25	<0.5	2.5	2.5	<5
T	OVT16	*Bacillus*	2.5	2.5	<0.5	<0.5	10	<5
T	OVT24	*Bacillus*	2.5	1.25	<0.5	<0.5	2.5	<5
L	OVL12	*Bacillus*	10	5	<0.5	1	<1	<5
T	OVT5	*Bacillus*	10	<0.5	<0.5	2.5	2.5	<5
F	OVF21	*Bacillus*	50	2.5	<0.5	10	2.5	<5
S	OVS24	*Bacillus*	>50	< 0.5	<0.5	5	2.5	<5
T	OVT10	*Bacillus*	50	<0.5	<0.5	5	2.5	<5
T	OVT20	*Bacillus*	50	5	<0.5	10	2.5	<5
L	OVL16	*Cellulosimicrobium*	>50	12.5	50	>50	10	<5
T	OVT9	*Chryseobacterium*	>50	>25	1	>50	>50	<5
S	OVS2	*Curtobacterium*	<0.5	2.5	2.5	>50	2.5	<5
S	OVS11	Curtobacterium	<0.5	12.5	50	>50	< 1	<5
S	OVS12	*Curtobacterium*	<0.5	12.5	50	>50	< 1	<5
S	OVS13	*Curtobacterium*	<0.5	2.5	50	50	<1	<5
S	OVS15	*Curtobacterium*	<0.5	12.5	50	>50	< 1	<5
L	OVL10	*Curtobacterium*	2.5	1.25	<0.5	10	<1	<5
S	OVS27	*Lysinibacillus*	>50	1.25	2.5	1	2.5	10
L	OVL14	*Microbacterium*	5	12.5	5	>50	< 1	<5
F	OVF19	*Micrococcus*	10	1.25	5	50	<1	<5
F	OVF24	*Micrococcus*	>50	1.25	5	50	<1	<5
F	OVF3	*Paenibacillus*	2.5	<0.5	<0.5	<0.5	2.5	10
F	OVF10	*Paenibacillus*	2.5	2.5	<0.5	<0.5	<1	<5
F	OVF2	*Pantoea*	10	2.5	<0.5	10	2.5	10
F	OVF14	*Pantoea*	10	2.5	<0.5	10	2.5	10
F	OVF1	*Pantoea*	>50	2.5	<0.5	10	2.5	10
F	OVF9	*Pantoea*	50	2.5	<0.5	10	2.5	10
F	OVF11	*Pantoea*	50	2.5	<0.5	5	2.5	10
F	OVF4	*Pseudomonas*	5	2.5	<0.5	2.5	25	<5
L	OVL17	*Pseudomonas*	5	2.5	<0.5	1	25	<5
S	OVS9	*Pseudomonas*	<0.5	2.5	<0.5	2.5	25	10
S	OVS14	*Pseudomonas*	<0.5	1.25	<0.5	<0.5	25	10
F	OVF7	*Pseudomonas*	2.5	<0.5	<0.5	<0.5	2.5	<5
F	OVF17	*Rathaybacter*	2.5	<0.5	2.5	10	<1	<5
F	OVF6	*Rhizobium*	5	2.5	<0.5	50	2.5	<5
S	OVS20	*Rhodococcus*	<0.5	2.5	<0.5	10	<1	<5
F	OVF18	*Rhodococcus*	2.5	2.5	<0.5	5	<1	<5
S	OVS7	*Sphingomonas*	<0.5	<0.5	1	<0.5	<1	<5
L	OVL6	*Sphingomonas*	50	< 0.5	<0.5	10	2.5	<5
S	OVS22	*Staphylococcus*	<0.5	1.25	1	>50	2.5	<5
T	OVT21	*Staphylococcus*	10	<0.5	<0.5	>50	2.5	<5
L	OVL18	*Xanthomonas*	50	5	<0.5	10	5	<5

*MIC values are reported as μg/ml. Str = Streptomycin, Tet = Tetracycline, Cip = Ciprofloxacin, Kan = Kanamicin, Chl = Chloramphenicol, Rif = Rifampicin. F = flower, L = leaf, S = stem, T = soil compartment.*

### Antagonistic Interaction Between *O. vulgare* Associated Bacteria

**FIGURE 4:**
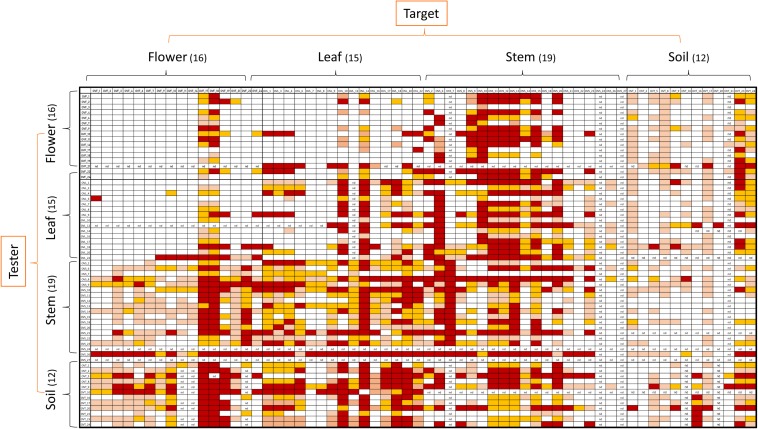
Heatmap showing the antagonistic interactions existing between endophytic (flower, leaf, stem) and soil bacteria isolated from the medicinal plant *O. vulgare*. Each strain was tested either as tester or target strain versus all the other ones. The inhibition values reflect three different inhibition levels observed during the cross-streak experiments, that is: complete (3, red), strong (2, orange), weak (1, salmon), and absence (0, white) of inhibition. Nd (not detected) refers to results that were not obtained.

Antagonistic interactions, at both inter- and intra-compartment level, were tested through cross-streak test, as described in Materials and Methods. To this purpose, each strain was used either as a tester or target. The antagonistic effect was indicated by the absence or reduction of the target strain growth. Data obtained, schematically shown in Figure 4, revealed that: (i) all the tested strains were able to inhibit at least one strain; (ii) soil-associated bacteria revealed a higher capacity to inhibit strains belonging to the other compartments, with those from stem and leaf following; (iii) flowers associated bacteria revealed the lowest capacity to inhibit other strains; (iv) regarding the inhibition sensitivity, the highest values were obtained from stem compartment, with leaf and flower following; and (v) the lowest inhibition sensitivity was revealed by soil-associated bacteria, as they were less inhibited by strains extracted from the different anatomical part of the plants.

**FIGURE 5:**
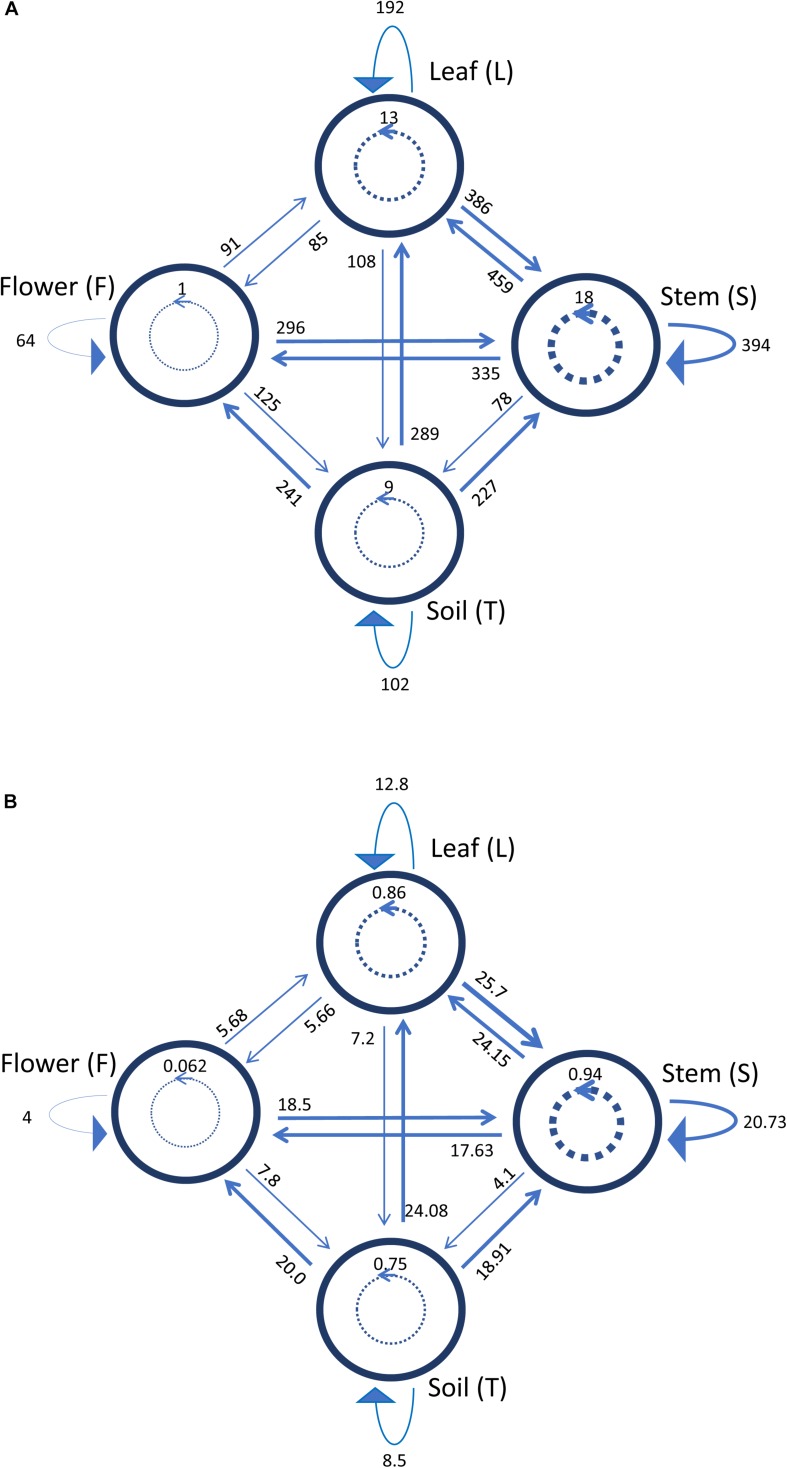
Schematic representation of inhibiting activity of bacterial strains isolated from the medicinal plant *O. vulgare* bulk soil (T), stem (S), leaf (L) and flowers (F). Each node represents a plant compartment whereas numbers represent the sum of the inhibiting scores of the bacteria isolated from those compartments **(A)** and the inhibition potential of the tester strains calculated as the sum of inhibition scores divided by the tester number **(B)**. Directed links represent inhibition patterns. Dashed links indicate the occurrence and the extent of self-inhibition.

Figure 5 shows a representation of the inhibitory pattern among strains from the same and different compartments. In this scheme, each node represents a plant compartment whereas numbers indicate the sum of the inhibiting scores of the bacteria isolated from those compartments and the inhibition potential of tester strains calculated as the sum of inhibition scores divided by the tester number. Inhibition patterns were represented by directed links, while the occurrence and the extent of self-inhibition were reported with dashed links.

The analysis of these results suggested that the bacterial communities from stem and soil were much more able to antagonize the growth of bacteria belonging to the other two compartments (Table 6). The relative degree of inhibitory score and sensitivity score of strains belonging to different compartments are respectively as following: soil > stem >leaves > flowers; soil < stem <flowers < leaf. Additionally, stem community showed the highest self-inhibition, whereas flower-associated bacteria revealed the lowest one. So, the different antagonistic activity observed could be, apparently, influenced by the ecological niche inhabited by bacteria.

**TABLE 6:** Inhibitory and sensitivity score of the endophytic bacteria associated to the four compartments of *O. vulgare*.

Compartment	Inhibitory score	Sensitivity score
Soil	63.41	20.25
Stem	47.62	47.99
Leaf	44.21	56.26
Flowers	32.27	46.39

### Inhibition of Human Pathogens by *O. vulgare* Associated Bacteria

The endophytic strains (Table 7) exhibiting high antagonistic interactions, have been further tested against human pathogens through cross-streak method, as described in Materials and Methods.

**TABLE 7:** *O. vulgare* associated bacteria tested against 46 pathogenic strains.

Compartment	Strain code	Taxonomy
Flower (F)	OVF 10	*Paenibacillus*
	OVF 22	*Bacillus*
	OVF11	*Pantoea*
	OVF 6	*Rhizobium*
	OVF 1	*Pantoea*
	OVF 24	*Micrococcus*
Leaf (L)	OVL 9	*Bacillus*
	OVL 1	*Arthrobacter*
	OVL 12	*Bacillus*
	OVL 18	*Xanthomonas*
Stem (S)	OVS 6	*Bacillus*
	OVS 8	*Arthrobacter*
	OVS26	*Bacillus*
	OVS 2	*Curtobacterium*
	OVS10	*Bacillus*
	OVS 23	*Arthrobacter*
	OVS 9	*Pseudomonas*
	OVS 18	*Arthrobacter*
	OVS 21	*Bacillus*
Soil (T)	OVT 9	*Chryseobacterium*
	OVT 1	*Agrobacterium*
	OVT 2	*Agromyces*
	OVT 10	*Bacillus*

**FIGURE 6:**
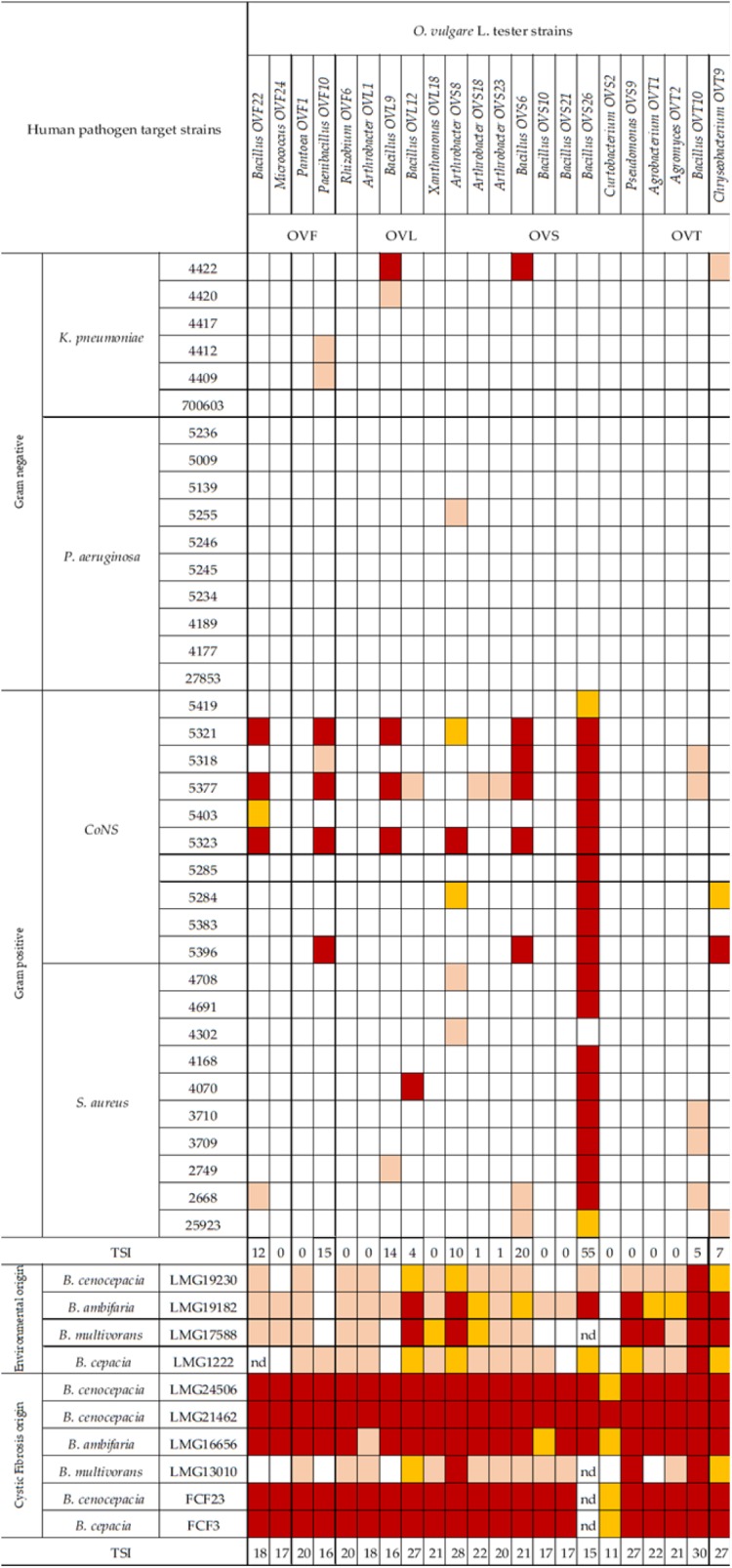
Antagonistic interactions of *O. vulgare* associated strains against human pathogenic strains. The inhibition values reflect three different inhibition levels observed during the cross-streak experiments, that is, complete (3, red), strong (2, orange), weak (1, salmon), and absence (0, white) of inhibition. Nd (not detected) refers to results that were not obtained. TSI refers to the total score of inhibition.

In Figure 6 the antagonistic interactions of *O. vulgare* tester strains against 46 human pathogens are shown.

**FIGURE 7:**
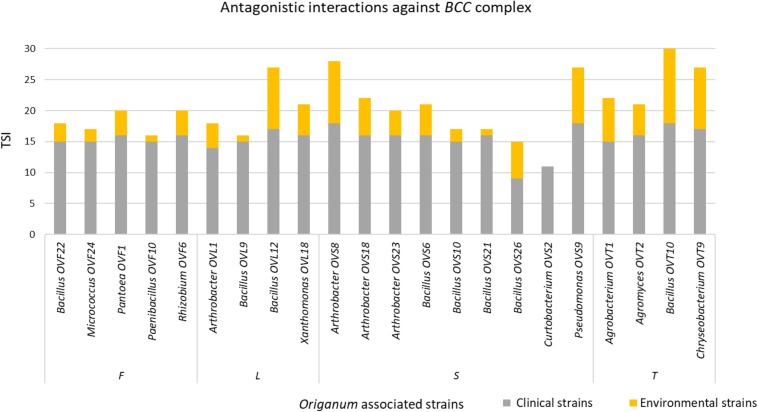
Antagonistic interactions of *O. vulgare* associated strains against *BCC* strains.

Interestingly, focusing on the antagonistic activity against BCC, *O. vulgare* strains were much more able to inhibit the growth of BCC members isolated from CF patients than those of environmental origins (Figure 7). All the flower testers exhibited a low total score of inhibition (TSI = 11–20), the 50% of leaves tester a strong TSI (TSI = 21–27), the 44.5% of stem tester a strong or very strong TSI (TSI = 21–27, 28–30), all the soil testers had a strong or very strong total score of inhibition (TSI = 21–27, 28–30).

**FIGURE 8:**
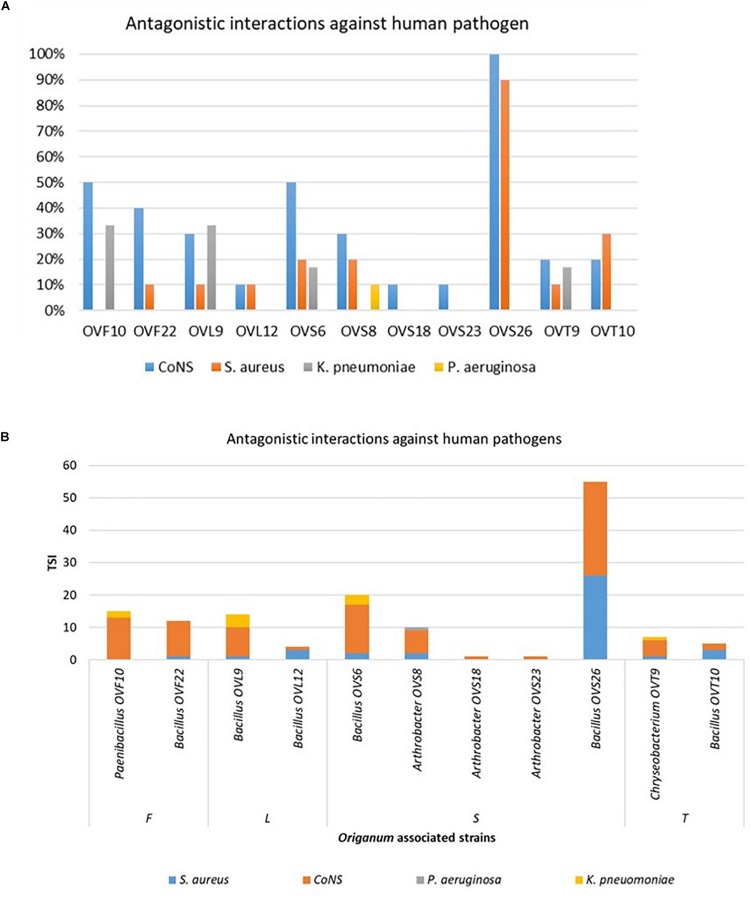
Antagonistic activity exhibited by endophytes strains against human pathogenic bacteria (*S. aureus*, CoNS, *P. aeruginosa, K. pneumoniae*) expressed as percentage of the antagonistic activity **(A)** and TSI **(B)**.

Focusing on the antagonistic activity against *S. aureus*, CoNS, *P. aeruginosa, K. pneumoniae*, among the 23 endophytes strains screened for antimicrobial activity, 11 (47.8%) showed antagonistic activity against at least one human pathogenic strain (Figure 6). This preliminary test demonstrated that 54.5% of the inhibitory strains belong to *Bacillus* genera, 27.3% to *Arthrobacter* and the remaining 18.2% to *Paenibacillus* and *Chryseobacterium* genera. Moreover, the interaction among inhibitory endophytes and pathogens gave better antagonistic results for Gram-positive bacteria (25.9%) than for Gram-negative bacteria (3.98%). The obtained data revealed that the major effect was recorded against the CoNS strains; the inhibitory interactions observed against this group of bacteria, indeed, were strong or complete in most cases (83.8%). In particular, the multidrug resistant strains *S. epidermidis* 5323, 5377 and 5321 were strongly inhibited by the endophytic isolates. Finally, stem-associated bacteria revealed the highest ability to inhibit pathogenic strains, while bulk soil associated bacteria revealed the lowest ability of inhibition. Figure 8A shows the percentage of the antagonistic activity exhibited by endophytes isolates against human pathogenic bacteria. OVS 26 showed broad spectrum of antagonistic activity against *S. aureus* and CoNS isolates (90% and 100%, respectively); the endophytes OVF 10, OVL 9, OVS 6 and OVT 9 showed the capacity to be active against four strains of *K. pneumoniae* (from 16.7 to 33.3%), while just OVS 8 was able to inhibit one *P. aeruginosa* strain. In general, all the eleven inhibitory endophytes showed degrees of antagonism against CoNS from 10 to 100%. Figure 8B shows the TSI of the antagonistic activity exhibited by endophytes strains against *S. aureus*, CoNS, *P. aeruginosa, K. pneumoniae* human pathogenic bacteria.

## Discussion and Conclusion

The potential of endophytes from medicinal plants to produce antibacterial, anticancer, and antifungal compounds is an emerging area in the last years (Miller et al., 2012; Maida et al., 2016; Chiellini et al., 2017; Maggini et al., 2018). *O. vulgare* is an aromatic and medicinal plant, whose EO has been extensively studied because of its diverse compositions among different specimens and remarkable characteristics (Lukas et al., 2015). Ecological and environmental effects, along with genetic variability, are some of the various reasons why EOs of *Origanum* species show major differences in their chemical composition (Vokou et al., 1993). The studied specimen, in particular, exhibited a germacrene D/β-caryophyllene chemotype, showing a predominance of the sesquiterpene hydrocarbon fraction. Other than the volatile compounds, the aerial parts of *O. vulgare* contain a remarkable variety of therapeutic bioactive compounds, including phenolic glycosides, flavonoids, tannins, sterols and high amounts of terpenoids (Pezzani et al., 2017).

The analysis of the total microbiota, obtained though NGS analysis, revealed that flower and stem compartments host a similar endophytic community, with a high diversity of genera; on the contrary, the leaf compartment appears to be very less diverse, containing only *Acinetobacter* genus.

The cultivable bacterial load in *O. vulgare* different districts ranged from about 10^2^ to 10^5^ CFU/g of soil, stem, leaf and flowers (fresh weight); these data are in agreement with previous reports on other different medicinal plant species (Emiliani et al., 2014; Checcucci et al., 2017).

The analysis of the structure of bacterial communities revealed that the dataset obtained with RAPD analysis highlights a high level of biodiversity at the strain level, especially regarding the anatomical parts of the plant. On the contrary, fingerprinting of the isolates associated to the bulk soil revealed a more clonal community. Moreover, a very low degree of sharing (3.1%) between the different compartments was revealed (only two haplotypes were shared between the leaf and the stem compartments), in agreement with data reported by Chiellini et al. (2014) about cultivable bacterial communities isolated from *Echinacea purpurea* (L.) Moench and *Echinacea angustifolia* (DC.).

The different genera identified through 16S rDNA gene amplification in *O. vulgare* plant have been detected in other plant species too, such as *E. purpurea* and *E. angustifolia* (Chiellini et al., 2014). In this work, *Bacillus* resulted to be the most represented genus and it was present in all districts analyzed, while other genera were exclusively present in specific ones. Overall, the RAPD and 16S rRNA gene analysis revealed that the selection of bacteria mainly occurs at the strain level, in agreement with previous data obtained with other medicinal plants, such as *Lavandula angustifolia* Mill. (Emiliani et al., 2014), *Echinacea purpurea* and *Echinacea angustifolia* (Chiellini et al., 2014; Maida et al., 2016). Therefore, different endophytic bacterial species are selected not only at the root or stem level, but also within plant tissues, as the different distribution of genera could suggest. Hence, it is possible that plant might “select” specific taxa to access its compartments.

In this work we have analyzed the antibiotic resistance profile of 62 cultivable bacterial strains isolated from different anatomical parts of *O. vulgare* plant and from the bulk soil. The obtained data revealed that: (i) most of the strains showed tolerance to at least the lowest concentrations of the antibiotic streptomycin and kanamycin used, and only a few strains were able to grow in presence of low concentrations of the antibiotic chloramphenicol and ciprofloxacin; a similar trend has already been evidenced in the case of *E. purpurea* (Mengoni et al., 2014); (ii) only a small number of strains (2 out of 19) tolerated the lowest concentrations of rifampicin; (iii) strains associated to the bulk soil resulted resistant against the great majority of the antibiotics tested, even at the highest concentrations used. The high tolerance to both streptomycin and kanamycin, shown by tested strains, could be due to similar resistance mechanisms. Moreover, the high resistance of strains associated to the bulk soil might be probably due to their exposure to commonly used antibiotics: in this scenario, their greater resistance to antibiotics could be explained.

Antagonistic inter and intra- compartment interactions were also evaluated. These analyses suggested that the bacterial communities from the stem and the soil compartments were particularly able to antagonize the growth of bacteria belonging to the other two compartments. Moreover, stem community revealed the highest self-inhibition levels, whereas flower-associated bacteria revealed the lowest one. Thus, the different antagonistic activity observed could be (apparently) influenced by the ecological niche inhabited by bacteria. Overall, the obtained results suggest that antagonistic interactions between bacterial strains might play a role in driving the diverse composition of the bacterial communities in the anatomical districts of the plants. It is important to stress that the used medium could influence the inhibitory patterns, and also that in different environmental conditions the inhibitory patterns could be different: for example, strains that do not show any inhibition on plates, *in vivo* could be stronger antagonists, and *vice versa*.

Strains that exhibited high inhibitory properties were tested against multidrug resistant human pathogens. Interestingly, regarding the antagonistic activity against BCC, *O. vulgare* strains were much more able to inhibit the growth of BCC members isolated from CF patients than those of environmental origins. On the other hand, 47.8% of *O. vulgare* strains showed antagonistic activity against at least one human pathogenic strain among *S. aureus*, Coagulase-negative staphylococci (CoNS), *P. aeruginosa* and *K. pneumoniae*, with the most active isolates displaying greater capacity of inhibition mainly against Gram-positive bacteria than Gram-negative ones. It has been proposed that, regarding Gram-negative bacteria, the presence of the outer polysaccharide membrane (made of various lipopolysaccharide constituents) could make them impermeable to lipophilic solutes (Silhavy et al., 2010; Naikpatil and Rathod, 2011).

At the same time, we can speculate that the different antimicrobial activity can depend on the different type of habitats that bacteria occupy: *Staphylococcus aureus* and CoNS are common colonizers of human epithelia, while *P. aeruginosa* and *K. pneumoniae* are opportunistic pathogens widely recovered from the environment. Hence, it is reasonable that bacteria capable of colonizing environmental habitats can exhibit intrinsic or acquired resistance against antimicrobial compounds produced by competing bacteria commonly found in the same ecological niches. Further studies should be performed to better understand this behavior.

In our opinion, data obtained in this work offer a preliminary but very promising example of the biotechnological potential of bacteria isolated from medicinal plants. In fact, the majority of the tested strains revealed a marked inhibitory effect between different compartments, widespread resistance to common antibiotics, and a strong capacity to inhibit the growth of MDR human pathogens. This last point is very important: nowadays research for new molecules active against human pathogens (MDR in particular) is one of the most important goals to achieve, mainly due to the increased resistance to commercially used antibiotics, an inevitable side effect of both the unsuitable use and over prescription of antibiotics in human medicine therapy and the antibiotics released into the environment (Helal et al., 2019). As known, medicinal plants and their associated microbiome represent a natural *reservoir* for bioactive compounds of pharmacological interest. Therefore, the bioactive compounds of selected strains could be purified and then tested for their bioactivity (or cytotoxicity) on human cultures and on animal models; it could be interesting to find out if the production of antimicrobial molecules in *O. vulgare* could be dependent on its associated microbiota. Furthermore, this study suggests that antagonistic interactions between bacterial strains might play a role in driving the diverse composition of the bacterial communities in the anatomical districts of the plants.

## Data Availability Statement

The datasets generated for this study can be found in the NCBI sequence read archive PRJNA606513, Gene Bank MN811044-MN811099.

## Author Contributions

RF, LP, AL, PB, and LC conceptualized the study and worked on the methodology. LC, CC, GB, and RA contributed to data curation and formal analysis. LC, SD, SC, AV, CC, IS, VD, RA, GB, and SB carried out the investigation. RF, LP, AL, and FF helped with project administration. FF was responsible for the resource. LC, SD, SC, AV, AL, CC, IS, VD, LP, RA, and GB validated the study. LC, CC, and RA wrote the original draft. LC, AV, SD, SC, CC, AL, VD, LP, RA, MD, VM, PB, AM, and RF reviewed and edited the manuscript.

## Conflict of Interest

The authors declare that the research was conducted in the absence of any commercial or financial relationships that could be construed as a potential conflict of interest.
