# Supplementary material for: The Cultivable Bacterial Microbiota Associated to the Medicinal Plant Origanum vulgare L.: From Antibiotic Resistance to Growth-Inhibitory Properties
Source: Front Microbiol. 2020 May 8;11:862. doi: 10.3389/fmicb.2020.00862 (PMC7226918; doi:10.3389/fmicb.2020.00862)
Supplement: Supplementary file 1 [file Data_Sheet_1.docx]

**Table S1.** Accession numbers of submitted 16S rDNA sequences. F=flower, L=leaf, S=stem, T= soil compartment.

| **Compartment** | **Code** | **GenBank A. No** | **Compartment** | **Code** | **GenBank A. No** |
| --- | --- | --- | --- | --- | --- |
|  |  |  |  |  |  |
| F | OVF1 | MN811044 | S | OVS2 | MN811075 |
| F | OVF2 | MN811044 | S | OVS6 | MN811076 |
| F | OVF3 | MN811045 | S | OVS7 | MN811077 |
| F | OVF4 | MN811046 | S | OVS8 | MN811078 |
| F | OVF6 | MN811047 | S | OVS9 | MN811079 |
| F | OVF7 | MN811048 | S | OVS10 | MN811080 |
| F | OVF9 | MN811049 | S | OVS11 | MN811075 |
| F | OVF10 | MN811050 | S | OVS12 | MN811075 |
| F | OVF11 | MN811051 | S | OVS13 | MN811075 |
| F | OVF14 | MN811052 | S | OVS14 | MN811081 |
| F | OVF17 | MN811053 | S | OVS15 | MN811075 |
| F | OVF18 | MN811054 | S | OVS18 | MN811082 |
| F | OVF19 | MN811055 | S | OVS20 | MN811083 |
| F | OVF21 | MN811056 | S | OVS21 | MN811084 |
| F | OVF22 | MN811057 | S | OVS22 | MN811085 |
| F | OVF24 | MN811058 | S | OVS23 | MN811086 |
| L | OVL1 | MN811059 | S | OVS24 | MN811087 |
| L | OVL3 | MN811060 | S | OVS26 | MN811088 |
| L | OVL4 | MN811061 | S | OVS27 | MN811089 |
| L | OVL6 | MN811062 | T | OVT1 | MN811090 |
| L | OVL 7 | MN811063 | T | OVT2 | MN811091 |
| L | OVL8 | MN811063 | T | OVT3 | MN811090 |
| L | OVL9 | MN811064 | T | OVT5 | MN811092 |
| L | OVL10 | MN811065 | T | OVT9 | MN811093 |
| L | OVL12 | MN811066 | T | OVT10 | MN811094 |
| L | OVL14 | MN811067 | T | OVT16 | MN811095 |
| L | OVL16 | MN811069 | T | OVT 17 | MN811090 |
| L | OVL17 | MN811070 | T | OVT20 | MN811096 |
| L | OVL18 | MN811071 | T | OVT21 | MN811097 |
| L | OVL20 | MN811073 | T | OVT23 | MN811098 |
| L | OVL22 | MN811074 | T | OVT24 | MN811099 |

**Table S2**. Complete composition of the studied *Origanum vulgare* L. essential oil

| **Compounds** | **l.r.i.^*^** | **Relative abundance (%)** |
| --- | --- | --- |
| α-thujene | 931 | 0.1 |
| α-pinene | 941 | 0.2 |
| sabinene | 976 | 6.4 |
| myrcene | 993 | 0.6 |
| α-terpinene | 1018 | 0.4 |
| *p*-cymene | 1027 | 0.4 |
| β-phellandrene | 1031 | 0.4 |
| 1,8-cineole | 1034 | 0.4 |
| (*Z*)-β-ocimene | 1042 | 4.0 |
| (*E*)-β-ocimene | 1052 | 3.6 |
| γ-terpinene | 1062 | 1.3 |
| terpinolene | 1088 | 0.2 |
| linalool | 1101 | 0.6 |
| 4-terpineol | 1178 | 2.4 |
| α-terpineol | 1189 | 0.4 |
| α-copaene | 1376 | 0.2 |
| β-bourbonene | 1384 | 2.8 |
| β-caryophyllene | 1420 | 19.2 |
| β-copaene | 1429 | 0.5 |
| aromadendrene | 1445 | 0.2 |
| *cis*-muurola-3,5-diene | 1447 | 0.1 |
| α-humulene | 1456 | 2.4 |
| *allo*aromadendrene | 1461 | 0.8 |
| *cis*-muurola-4(14),5-diene | 1462 | 0.8 |
| γ-muurolene | 1477 | 0.2 |
| germacrene D | 1478 | 29.4 |
| bicyclogermacrene | 1496 | 5.5 |
| α-muurolene | 1498 | 0.7 |
| germacrene A | 1506 | 0.7 |
| (*E*,*E*)-α-farnesene | 1507 | 5.5 |
| δ-cadinene | 1524 | 4.0 |
| (E)-α-bisabolene | 1531 | 0.5 |
| α-cadinene | 1538 | 0.1 |
| germacrene D-4-ol | 1575 | 0.8 |
| spathulenol | 1576 | 1.1 |
| caryophyllene oxide | 1581 | 1.5 |
| globulol | 1583 | 0.2 |
| *epi*-α-cadinol | 1640 | 0.6 |
| α-cadinol | 1654 | 0.7 |
| Monoterpene hydrocarbons |  | 17.6 |
| Oxygenated monoterpenes |  | 3.7 |
| Sesquiterpene hydrocarbons |  | 73.5 |
| Oxygenated sesquiterpenes |  | 4.8 |
| Total identified (%): |  | 99.7 |
| ^*^ Linear retention indices on a DB-5 capillary column. | | |

**Table S3**: Distribution of the RAPD haplotypes, 16S gene sequence accession number and taxonomy of bacteria associated to the medicinal plant *Origanum vulgare* L..

| **Haplotype** | **Flower (OVF)** | **Leaf**  **(OVL)** | **Stem**  **(OVS)** | **Soil**  **(OVT)** | **N. isolates** | **Accession**  **number** | **Taxonomy** |
| --- | --- | --- | --- | --- | --- | --- | --- |
| **1** | 1; 5 |  |  |  | 2 | MN811044 | *Pantoea* |
| **2** | 2 |  |  |  | 1 | MN811044 | *Pantoea* |
| **3** | 3 |  |  |  | 1 | MN811045 | *Paenibacillus* |
| **4** | 4;8;15 |  |  |  | 3 | MN811046 | *Pseudomonas* |
| **5** | 6;12;13 |  |  |  | 3 | MN811047 | *Rhizobium* |
| **6** | 7 |  |  |  | 1 | MN811048 | *Pseudomonas* |
| **7** | 9 |  |  |  | 1 | MN811049 | *Pantoea* |
| **8** | 10 |  |  |  | 1 | MN811050 | *Paenibacillus* |
| **9** | 11 |  |  |  | 1 | MN811051 | *Pantoea* |
| **10** | 14 |  |  |  | 1 | MN811052 | *Pantoea* |
| **11** | 16;18 |  |  |  | 2 | MN811054 | *Rhodococcus* |
| **12** | 17 |  |  |  | 1 | MN811053 | *Rathaybacter* |
| **13** | 19; 20; 23 |  |  |  | 3 | MN811055 | *Micrococcus* |
| **14** | 21 |  |  |  | 1 | MN811056 | *Bacillus* |
| **15** | 22 |  |  |  | 1 | MN811057 | *Bacillus* |
| **16** | 24 |  |  |  | 1 | MN811058 | *Micrococcus* |
| **17** |  | 1;2;23; |  |  | 3 | MN811059 | *Arthrobacter* |
| **18** |  | 3 |  |  | 1 | MN811060 | *Arthrobacter* |
| **19** |  | 4 |  |  | 1 | MN811061 | *Arthrobacter* |
| **20** |  | 5;10;13;21; |  |  | 4 | MN811065 | *Curtobacterium* |
| **21** |  | 6 |  |  | 1 | MN811062 | *Sphingomonas* |
| **22** |  | 7 |  |  | 1 | MN811063 | *Bacillus* |
| **23** |  | 8 |  |  | 1 | MN811063 | *Bacillus* |
| **24** |  | 9;11 |  |  | 2 | MN811064 | *Bacillus* |
| **25** |  | 12 |  |  | 1 | MN811066 | *Bacillus* |
| **26** |  | 14 |  |  | 1 | MN811067 | *Microbacterium* |
| **27** |  | 15;24 | 6 |  | 3 | MN811076 | *Bacillus* |
| **28** |  | 16 |  |  | 1 | MN811069 | *Cellulosimicrobium* |
| **29** |  | 17 |  |  | 1 | MN811070 | *Pseudomonas* |
| **30** |  | 18 |  |  | 1 | MN811071 | *Xanthomonas* |
| **31** |  | 19 | 25;26 |  | 3 | MN811088 | *Bacillus* |
| **32** |  | 20 |  |  | 1 | MN811073 | *Arthrobacter* |
| **33** |  | 22 |  |  | 1 | MN811074 | *Arthrobacter* |
| **34** |  |  | 2;3;4; |  | 3 | MN811075 | *Curtobacterium* |
| **35** |  |  | 5;8 |  | 2 | MN811078 | *Arthrobacter* |
| **36** |  |  | 7 |  | 1 | MN811077 | *Sphingomonas* |
| **37** |  |  | 9 |  | 1 | MN811079 | *Pseudomonas* |
| **38** |  |  | 10 |  | 1 | MN811080 | *Bacillus* |
| **39** |  |  | 11 |  | 1 | MN811075 | *Curtobacterium* |
| **40** |  |  | 12 |  | 1 | MN811075 | *Curtobacterium* |
| **41** |  |  | 13 |  | 1 | MN811075 | *Curtobacterium* |
| **42** |  |  | 14 |  | 1 | MN811081 | *Pseudomonas* |
| **43** |  |  | 15;16 |  | 2 | MN811075 | *Curtobacterium* |
| **44** |  |  | 18;19 |  | 2 | MN811082 | *Arthrobacter* |
| **45** |  |  | 20 |  | 1 | MN811083 | *Rhodococcus* |
| **46** |  |  | 21 |  | 1 | MN811084 | *Bacillus* |
| **47** |  |  | 22 |  | 1 | MN811085 | *Staphylococcus* |
| **48** |  |  | 23 |  | 1 | MN811086 | *Arthrobacter* |
| **49** |  |  | 24 |  | 1 | MN811087 | *Bacillus* |
| **50** |  |  | 27 |  | 1 | MN811089 | *Lysinibacillus* |
| **51** |  |  |  | 1 | 1 | MN811090 | *Agrobacterium* |
| **52** |  |  |  | 2;4;7;11;13;18;22; | 7 | MN811091 | *Agromyces* |
| **53** |  |  |  | 3;6;8;12;14; | 5 | MN811090 | *Agrobacterium* |
| **54** |  |  |  | 5 | 1 | MN811092 | *Bacillus* |
| **55** |  |  |  | 9;15 | 2 | MN811093 | *Chryseobacterium* |
| **56** |  |  |  | 10 | 1 | MN811094 | *Bacillus* |
| **57** |  |  |  | 16 | 1 | MN811095 | *Bacillus* |
| **58** |  |  |  | 17;19 | 2 | MN811090 | *Agrobacterium* |
| **59** |  |  |  | 20 | 1 | MN811096 | *Bacillus* |
| **60** |  |  |  | 21 | 1 | MN811097 | *Staphylococcus* |
| **61** |  |  |  | 23 | 1 | MN811098 | *Arthrobacter* |
| **62** |  |  |  | 24 | 1 | MN811099 | *Bacillus* |
| **N. isolates** | **24** | **24** | **25** | **24** | **97** |  |  |
| **N. haplotypes** | **16** | **17** | **19** | **12** | **-** |  |  |


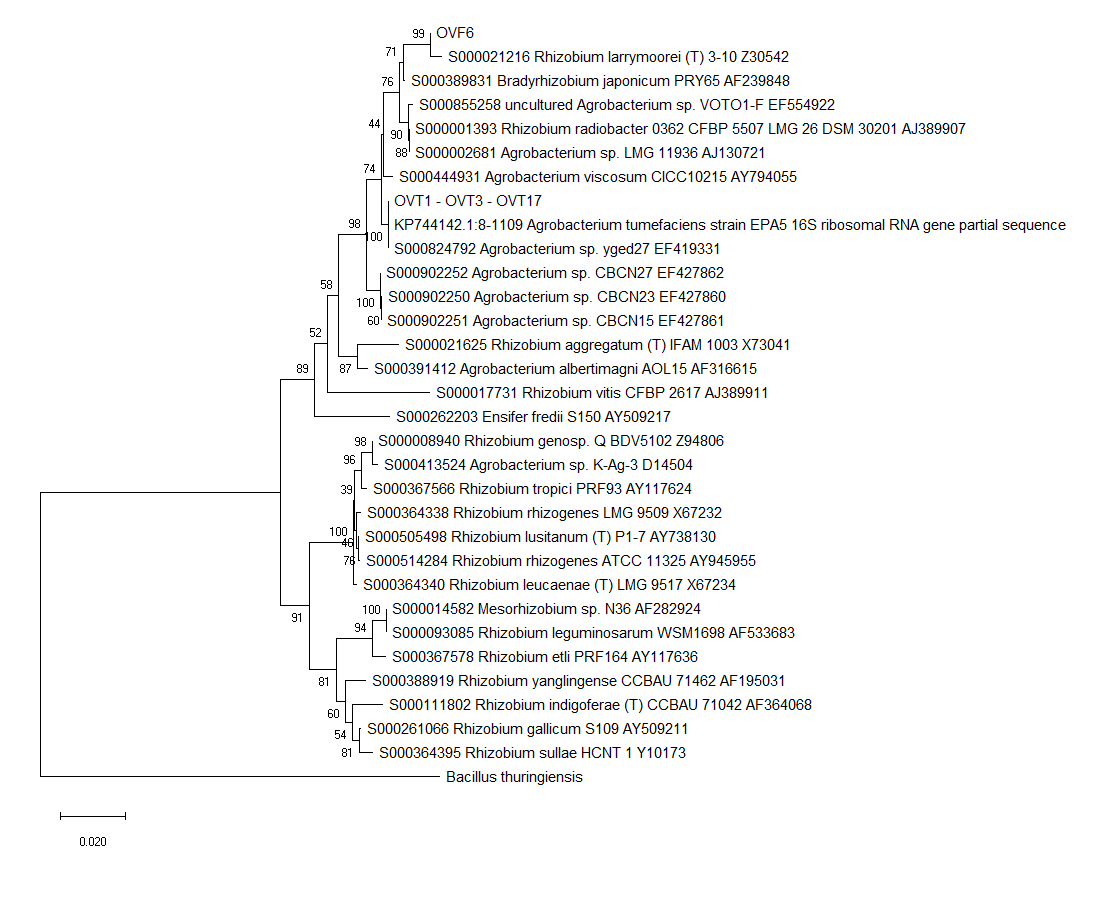


**Figure S1.** Phylogenetic tree for the genera *Rhizobium* and *Agrobacterium.*


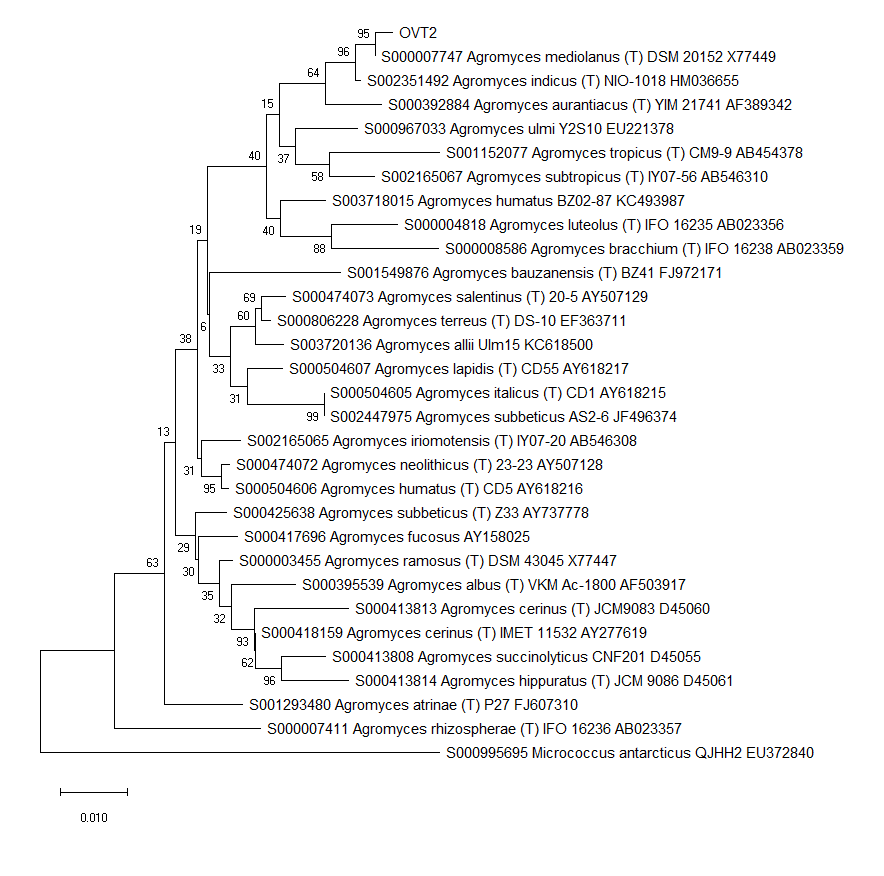


**Figure S2.** Phylogenetic tree for the genus *Agromyces.*


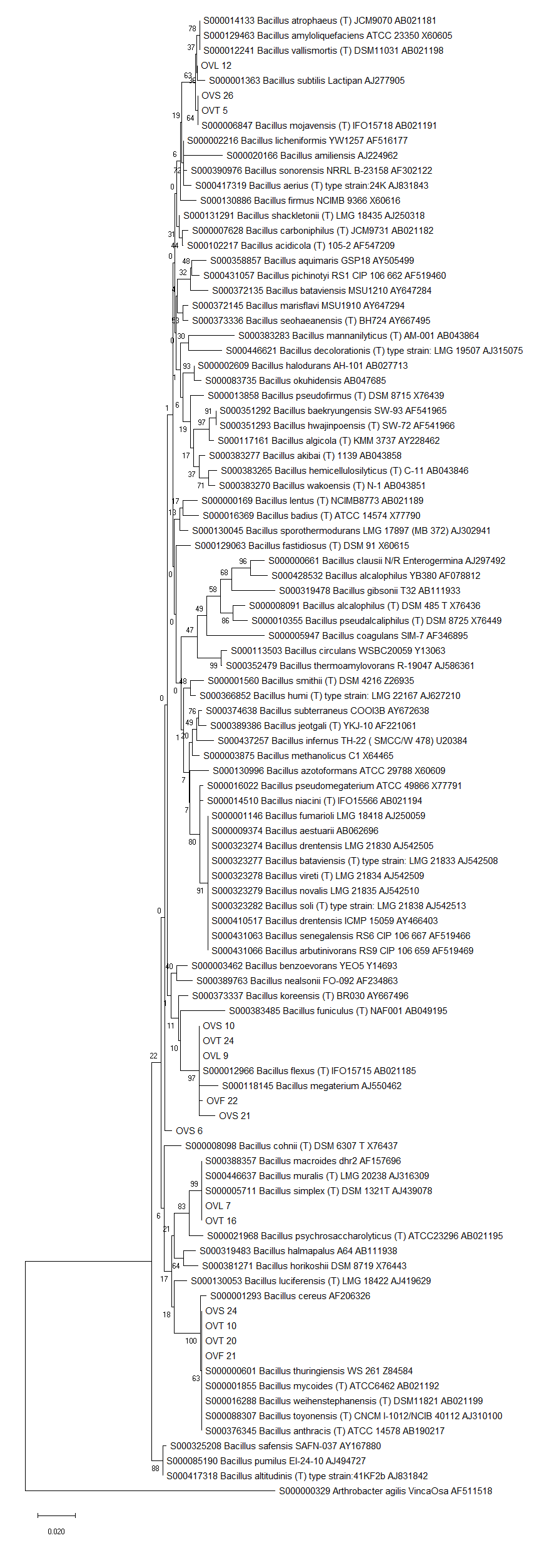


**Figure S3.** Phylogenetic tree for the genus *Bacillus*.


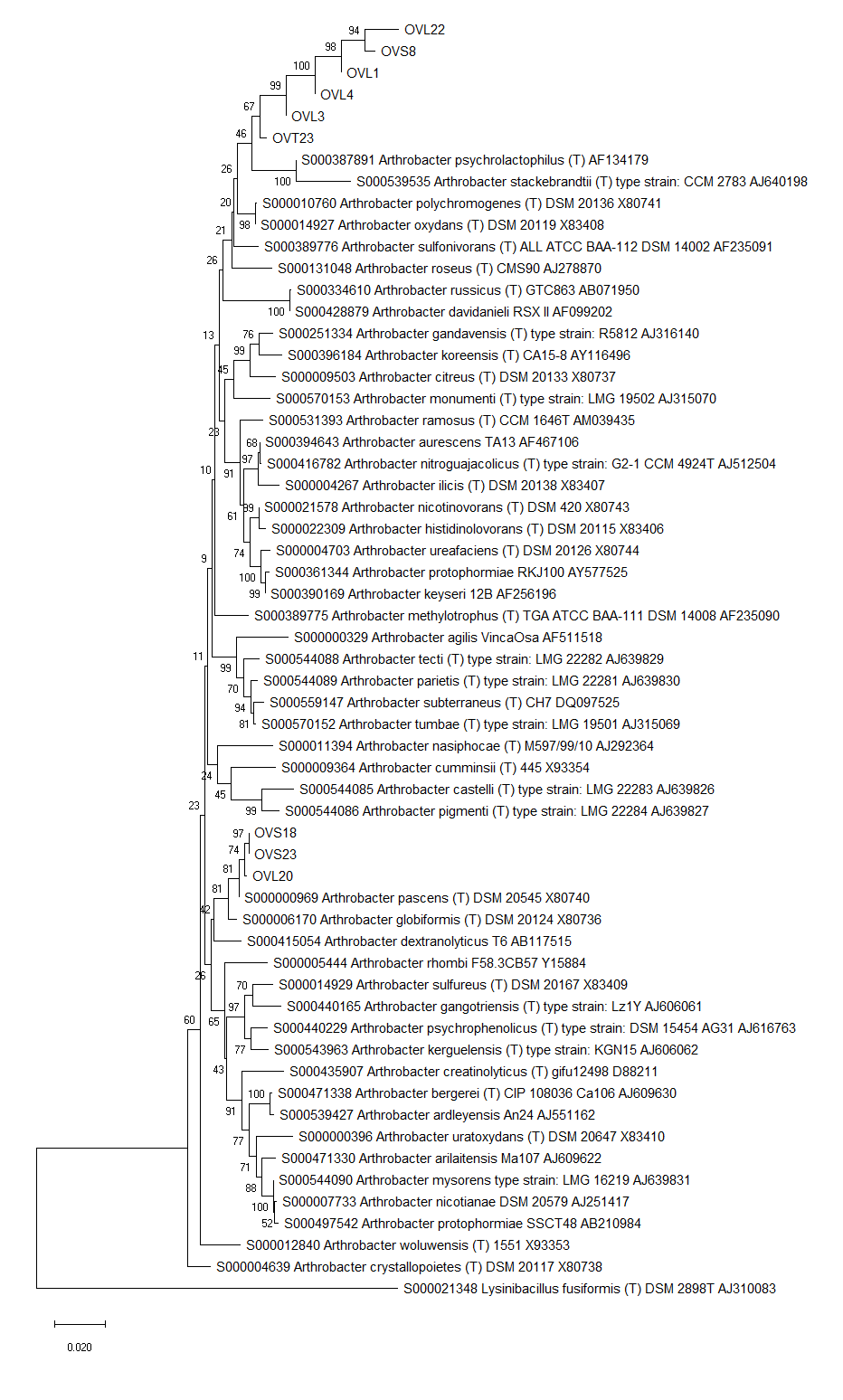


**Figure S4.** Phylogenetic tree for the genus *Arthrobacter.*


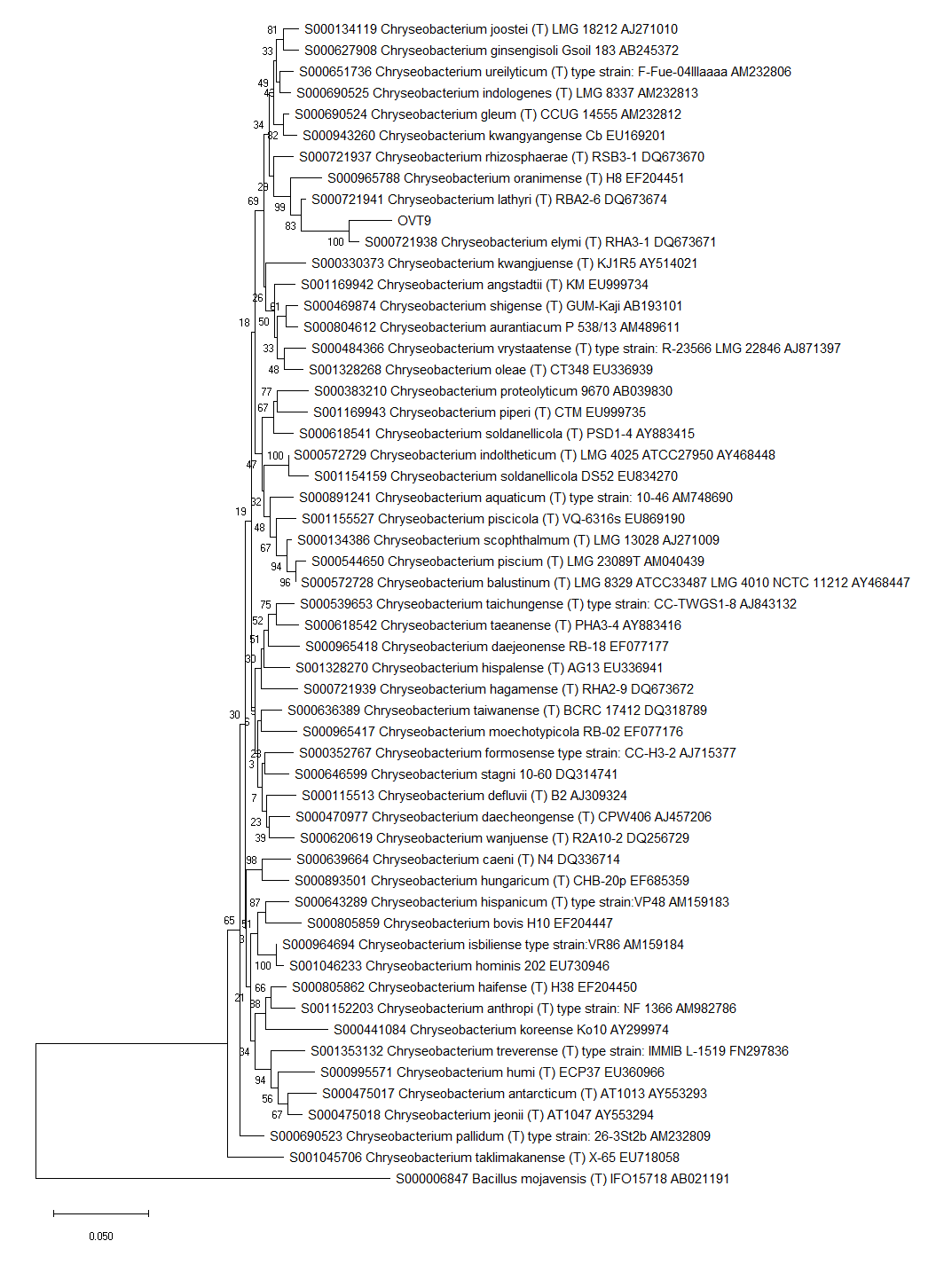
 **Figure S5.** Phylogenetic tree for the genus *Chryseobacterium.*


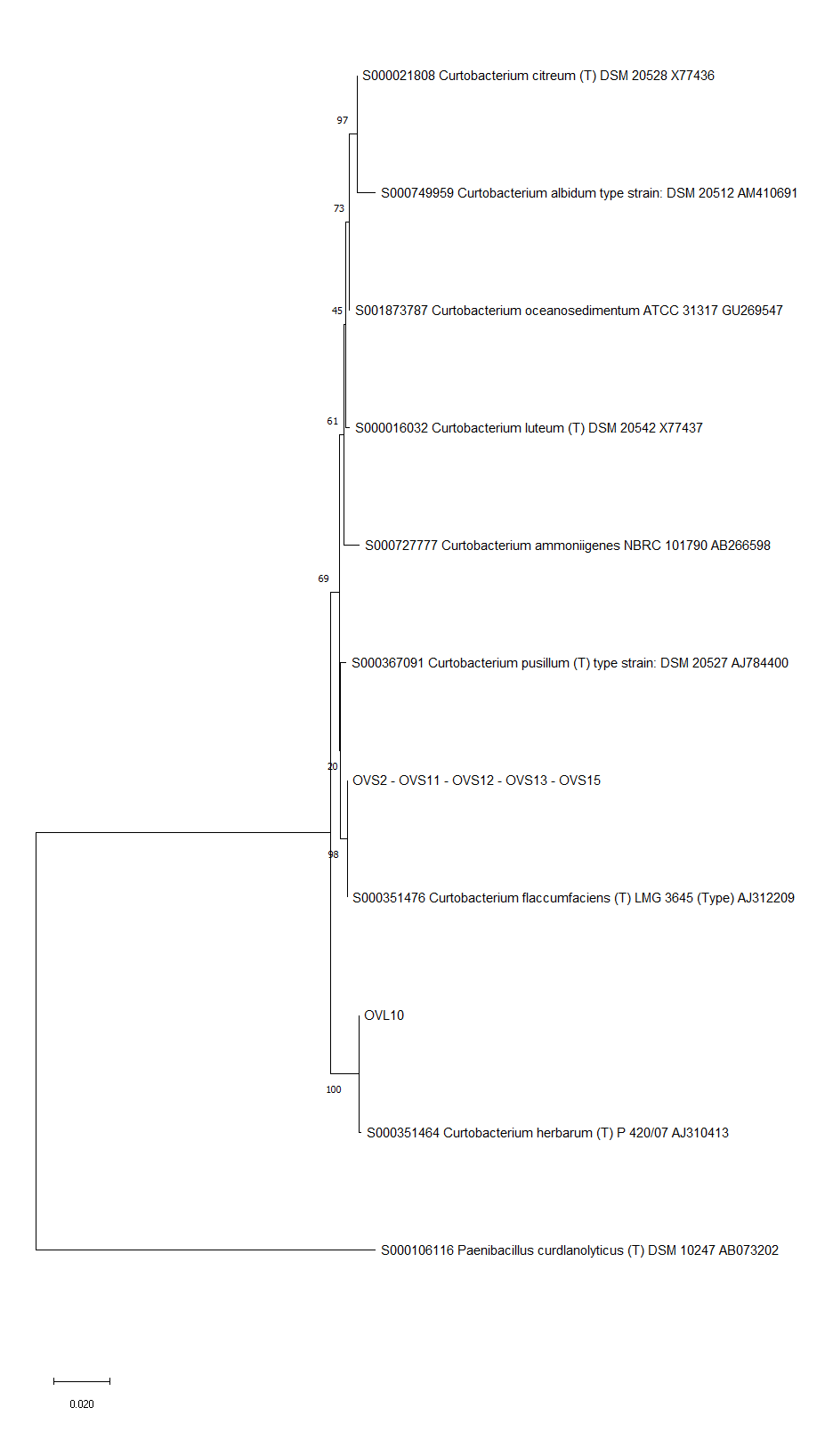


**Figure S6.** Phylogenetic tree for the genus *Curtobacterium.*


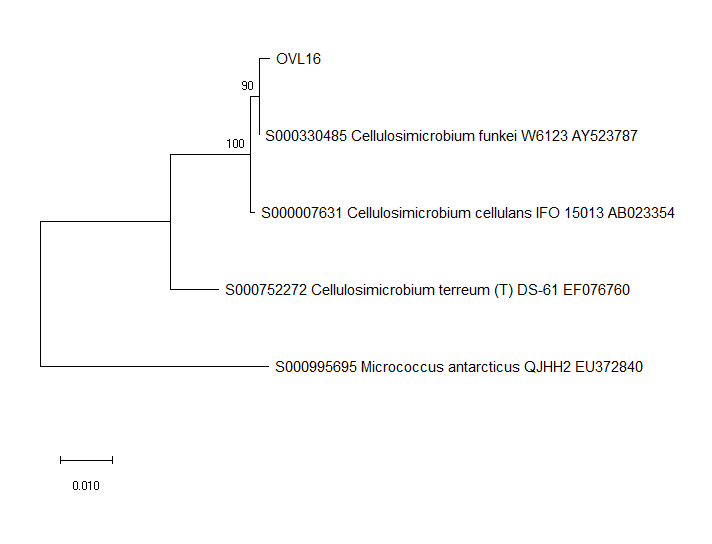


**Figure S7**. Phylogenetic tree for the genus *Cellulosimicrobium.*


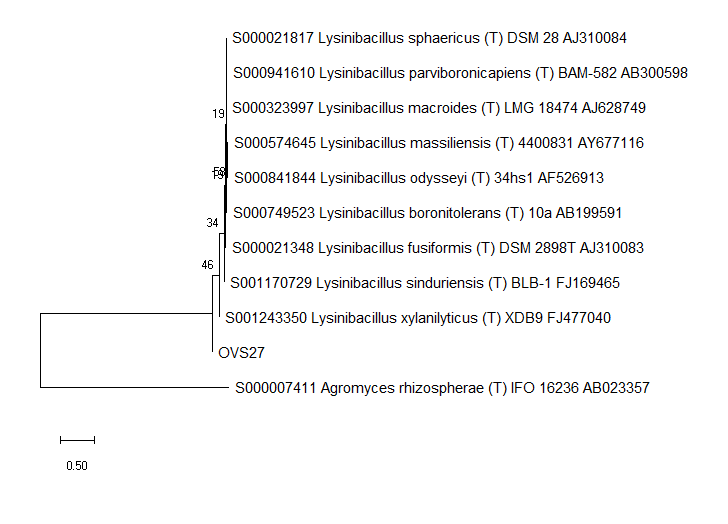


**Figure S8.** Phylogenetic tree for the genus *Lysinibacillus.*


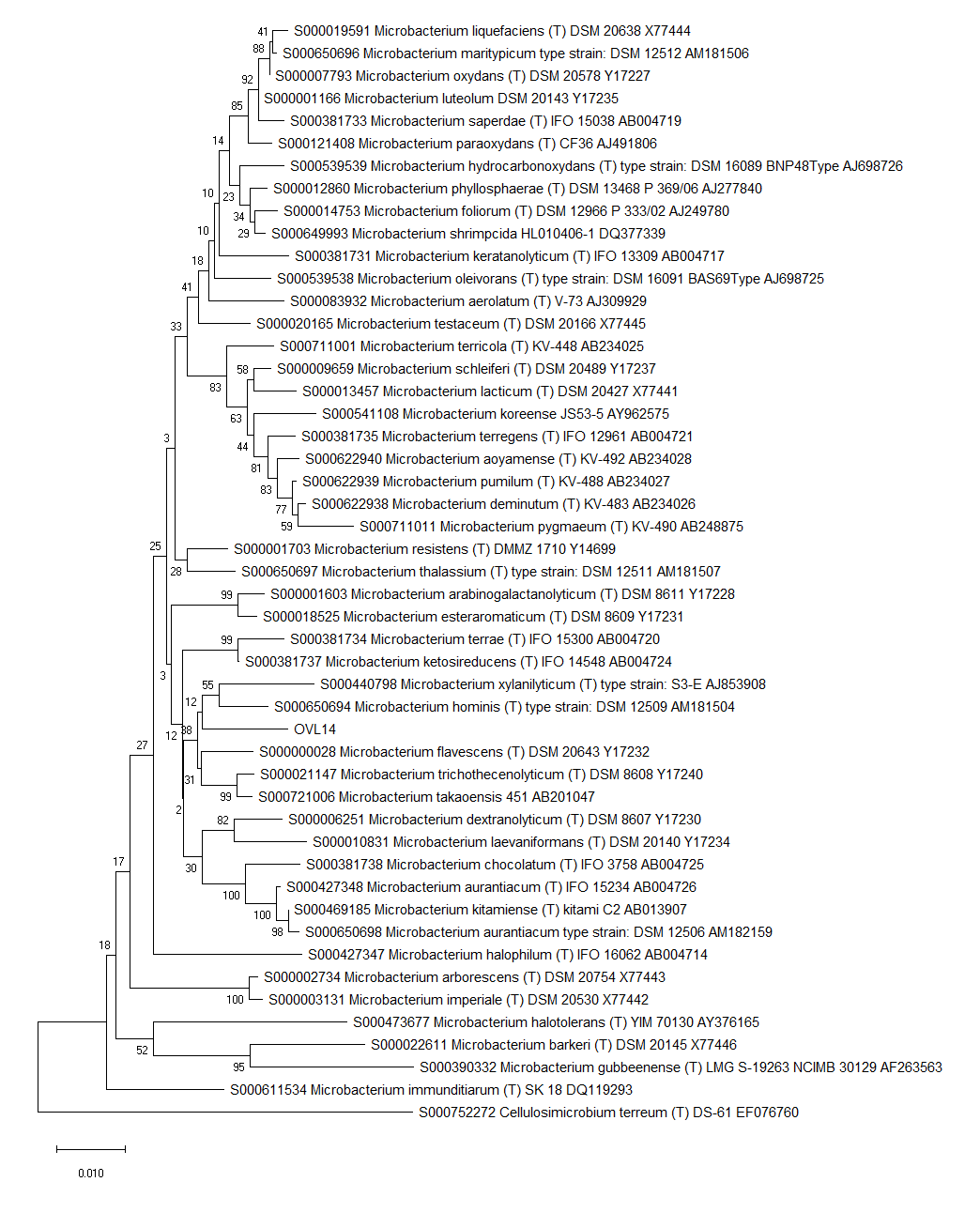


**Figure S9.** Phylogenetic tree for the genus *Microbacterium.*


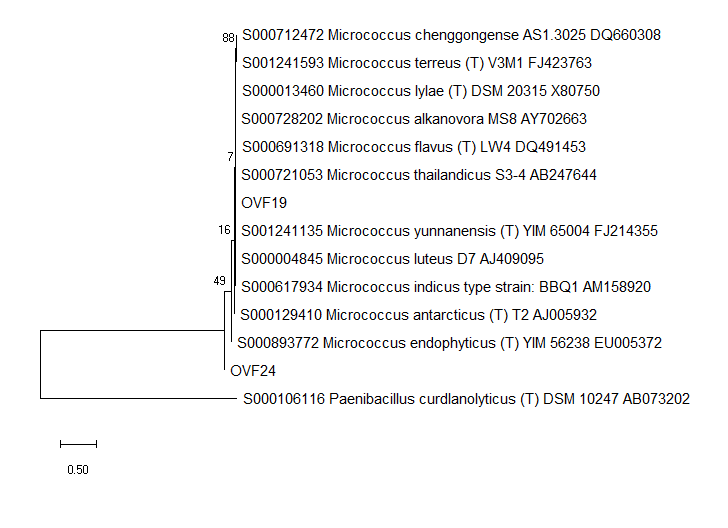


**Figure S10.** Phylogenetic tree for the genus *Micrococcus.*


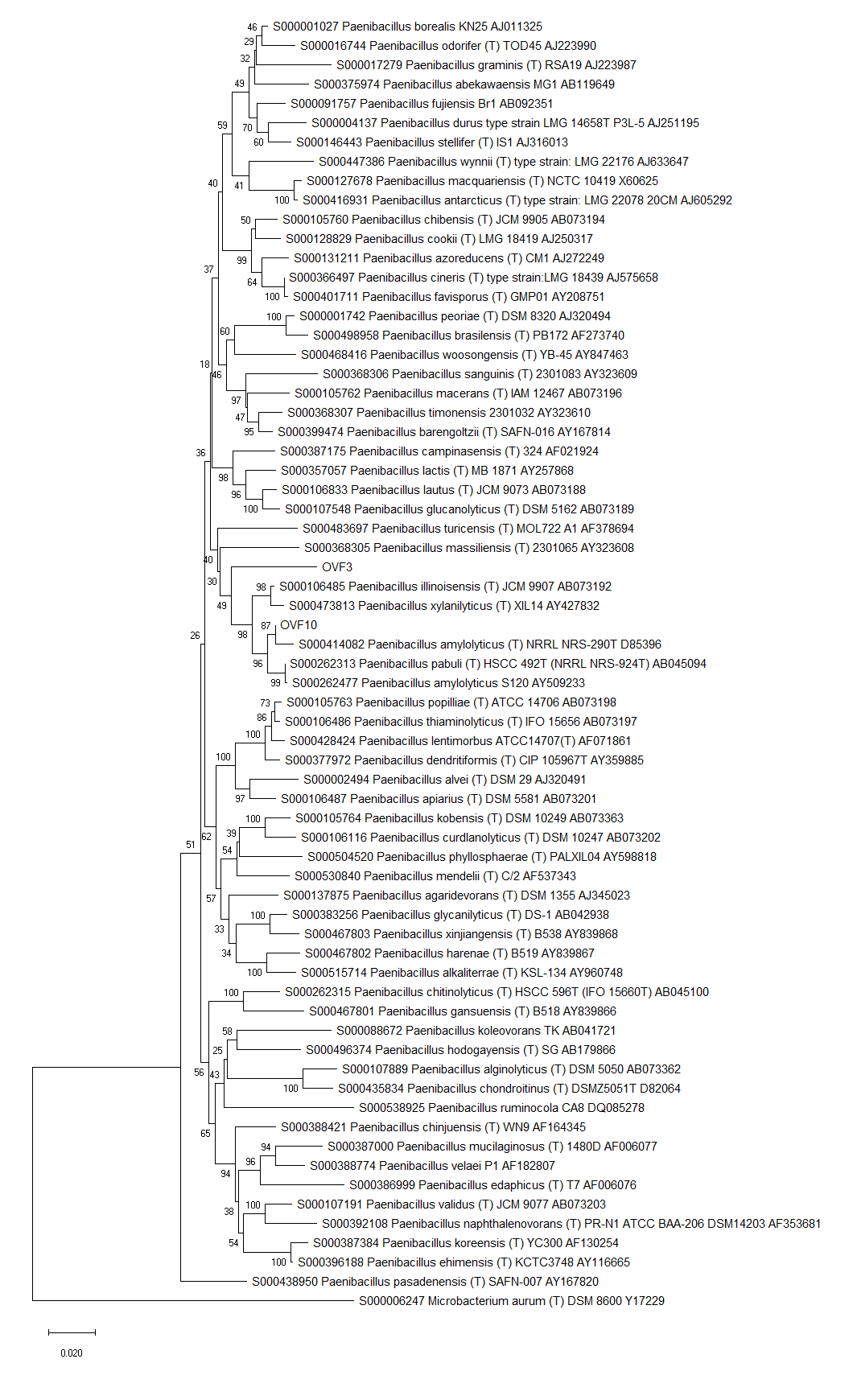


**Figure S11.** Phylogenetic tree for the genus *Paenibacillus.*


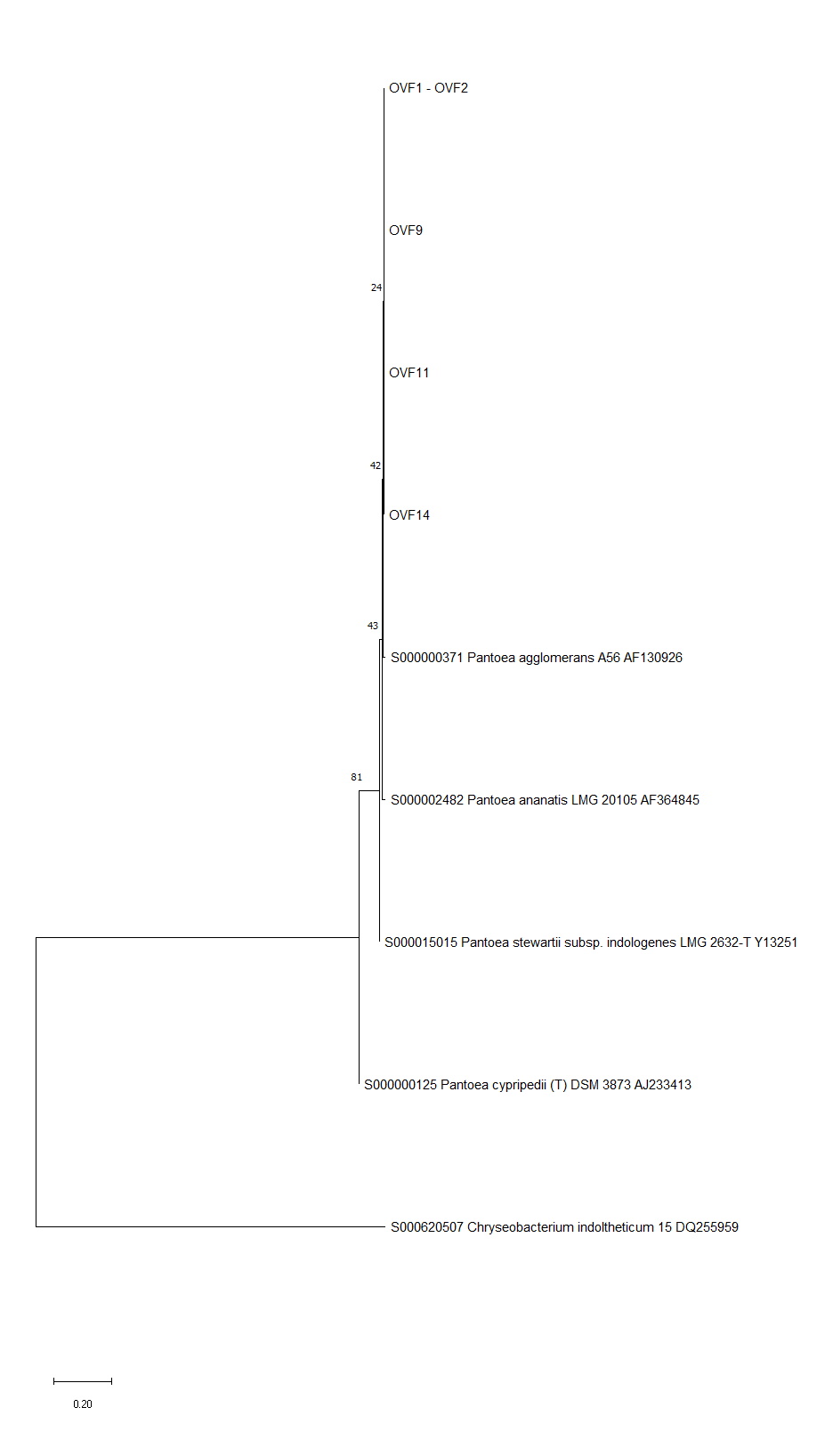


**Figure S12.** Phylogenetic tree for the genus *Pantoea.*


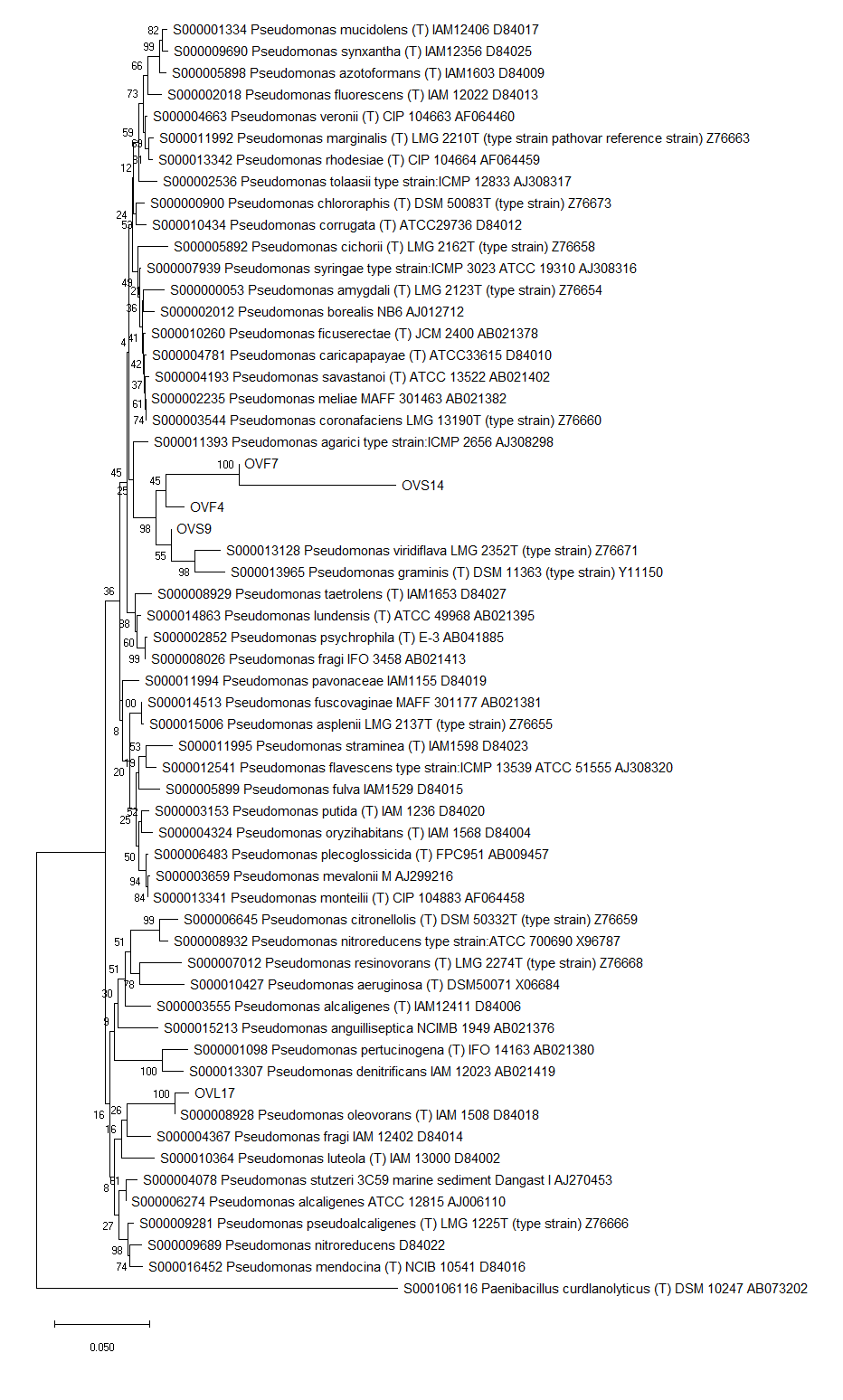


**Figure S13.** Phylogenetic tree for the genus *Pseudomonas.*


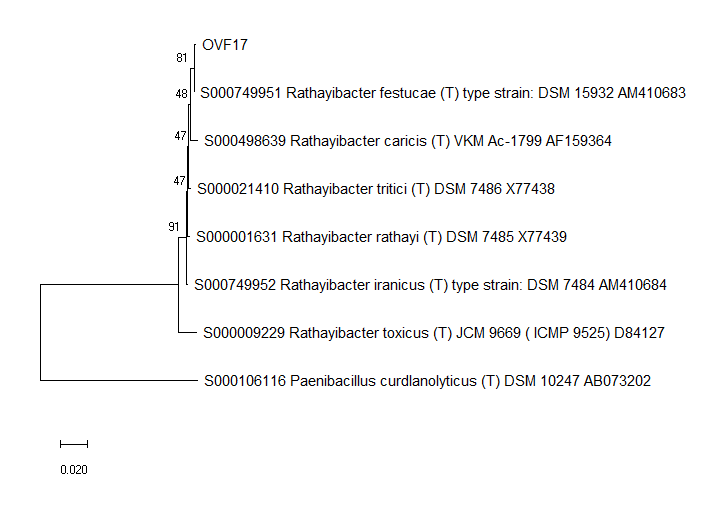


**Figure S14.** Phylogenetic tree for the genus *Rathaybacter.*


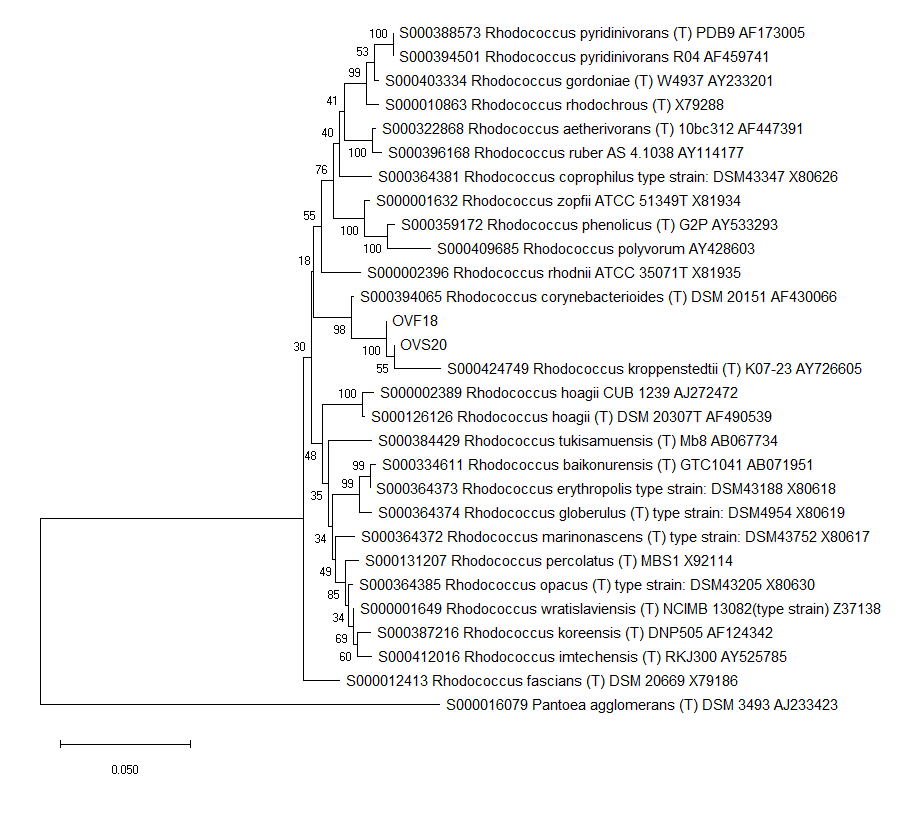


**Figure S15.** Phylogenetic tree for the genus *Rhodococcus.*


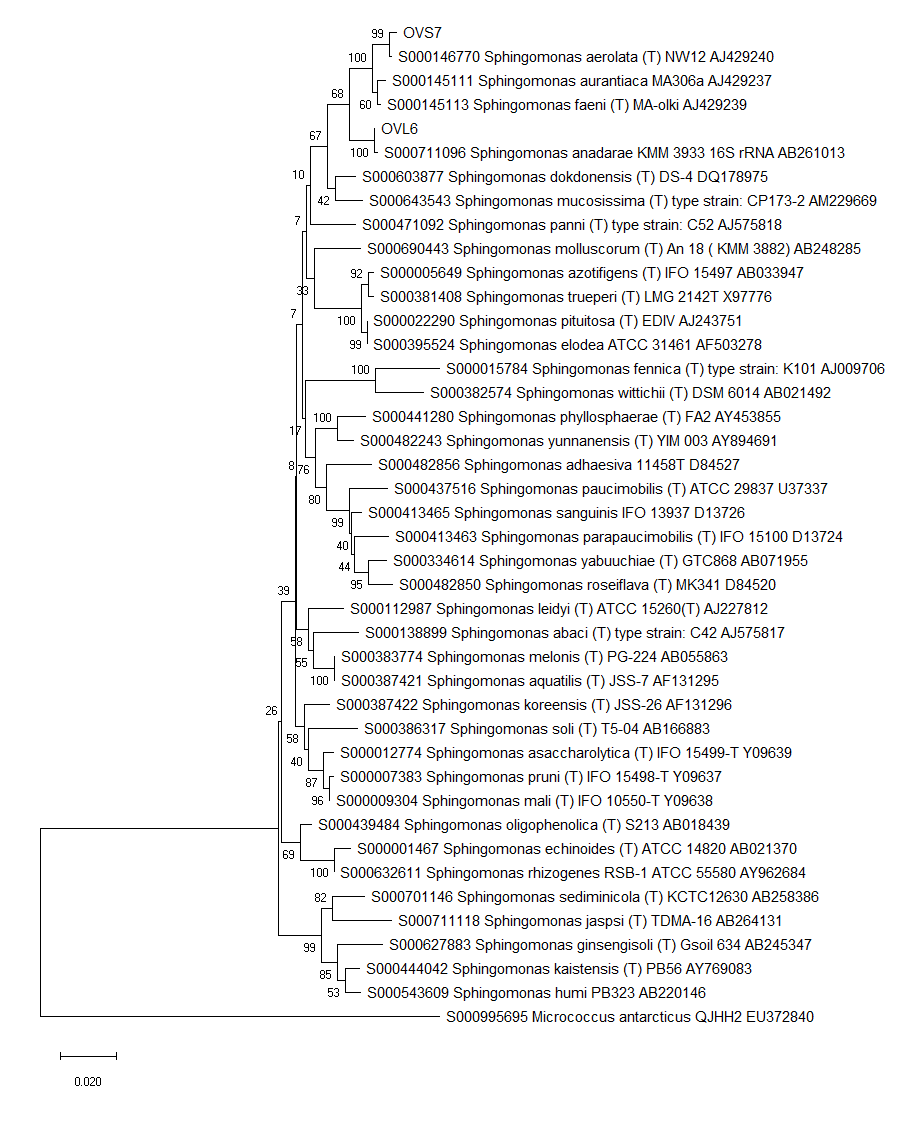


**Figure S16.** Phylogenetic tree for the genus *Sphingomonas.*


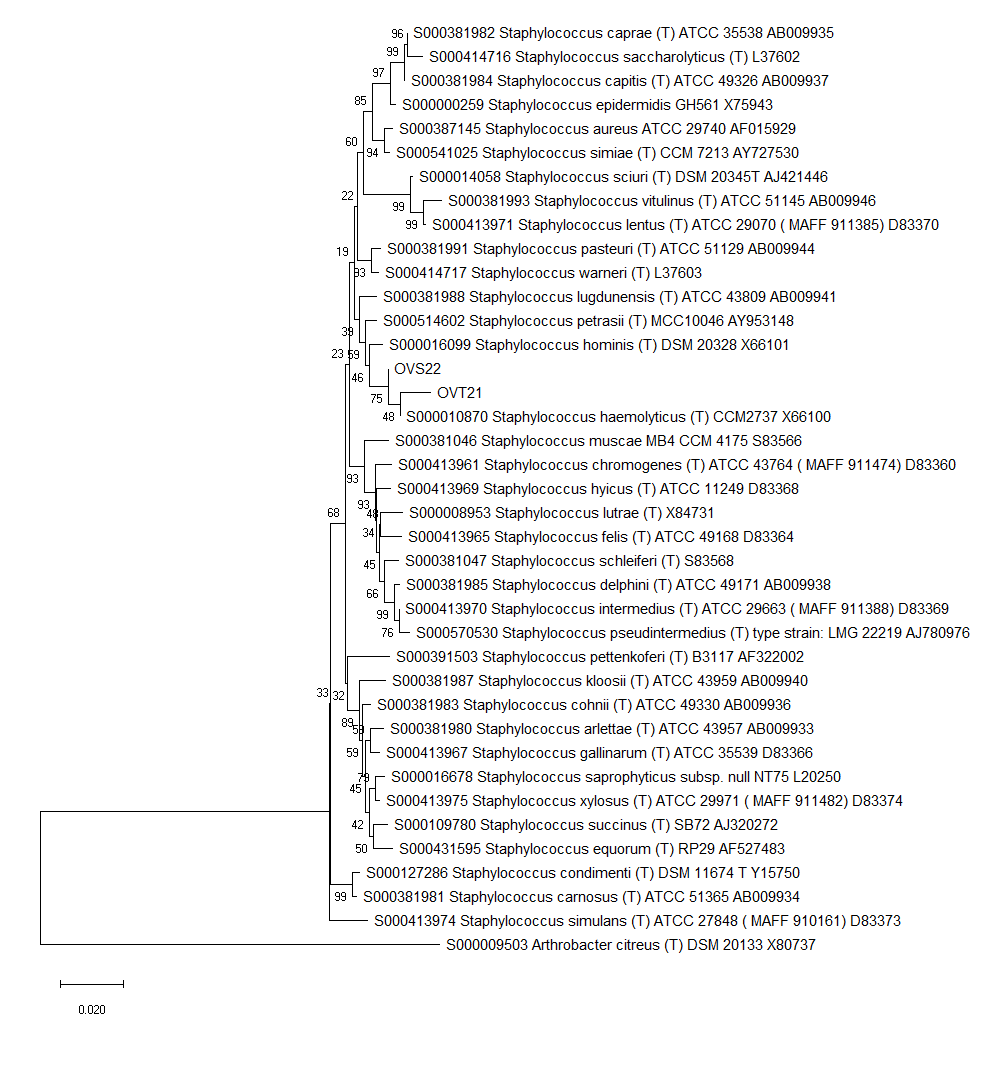


**Figure S17.** Phylogenetic tree for the genus *Staphylococcus.*


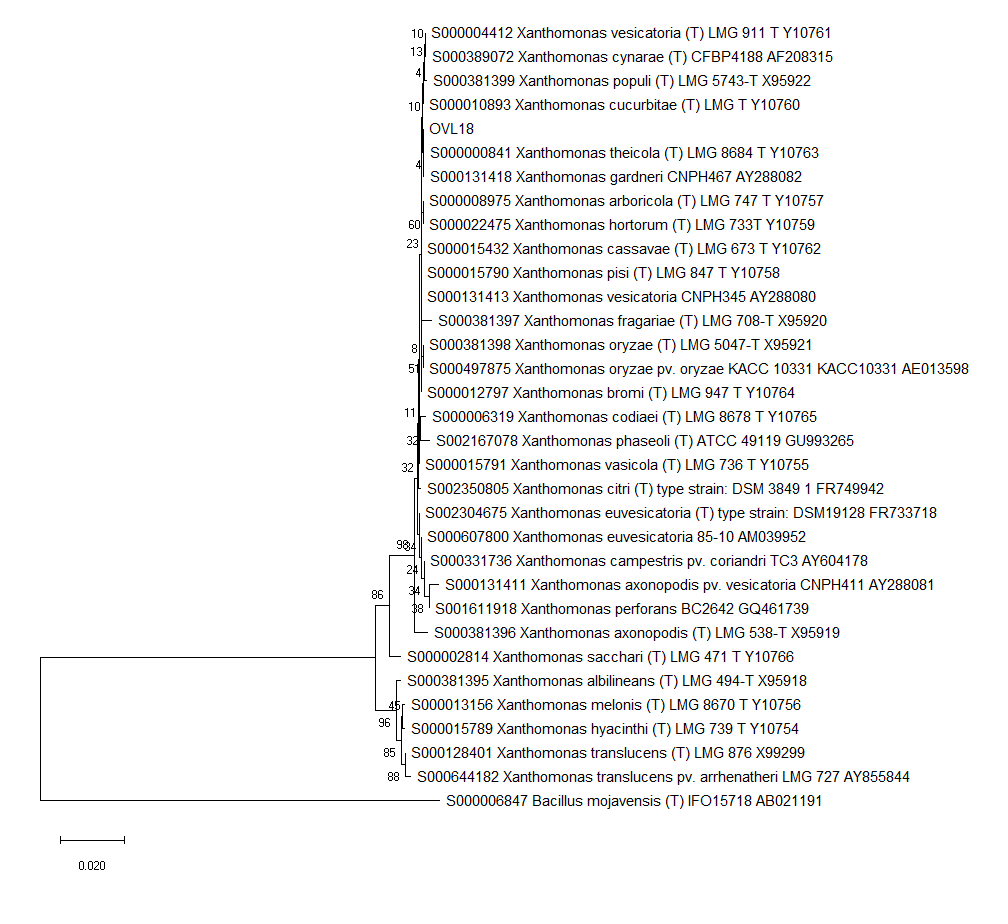


**Figure S18.** Phylogenetic tree for the genus *Xanthomonas.*
